# The Impact of Tax Culture on Tax Rate Structure Preferences: Results from a Vignette Study with Migrants and Non-Migrants in Germany

**DOI:** 10.1515/rle-2024-0041

**Published:** 2025-06-06

**Authors:** Dirk Kiesewetter, André Machwart

**Affiliations:** School of Business and Economics, Department of Business Management and Business Taxation, Julius-Maximilians-Universität Würzburg, Sanderring 2, D-97070 Würzburg, Germany

**Keywords:** migration, tax culture, tax rate progressivity, tax rate structure preferences, vignette approach, H24, H3, C90

## Abstract

We explore the relationship between tax culture and tax rate structure preferences among migrants and non-migrants in Germany. A vignette study is used to examine (1) whether migrants bring their country of origin’s tax culture to the destination country and (2) whether second-generation migrants assimilate with the host society’s tax culture. Our findings provide evidence for the impact of tax culture. Migrants tend to prefer a less-progressive tax rate structure, especially those from flat tax countries. Additionally, while second-generation migrants align their preferences with those of the host society, differences remain. This research provides insights into the dynamics of tax culture in heterogeneous societies.

## List of abbreviations

Max: maximum expression
MB participants: participants with a migration background
MB FLAT participants: participants with a flat tax migration background (subsample of migrant participants)
MB FLAT 1st GEN participants: participants with a flat tax migration background of the first generation (subsample of migrant participants)
MB FLAT 2nd GEN participants: participants with a flat tax migration background of the second generation (subsample of migrant participants)
MB NO FLAT participants: participants without a flat tax migration background (subsample of migrant participants)
MB NO FLAT 1st GEN participants: participants without a flat tax migration background of the first generation (subsample of migrant participants)
MB NO FLAT 2nd GEN participants: participants without a flat tax migration background of the second generation (subsample of migrant participants)
MB 1st GEN participants: participants with a first-generation migration background (subsample of migrant participants)
MB 2nd GEN participants: participants with a second-generation migration background (subsample of migrant participants)
Min.: minimum expression
N: number of observations
NO MB: participants without a migration background
OLS: ordinary least square
SD: standard deviation

## 1 Introduction

Personal income taxation serves as a primary means of funding the state budget. In 2021, income tax in Organization for Economic Co-operation and Development (OECD) countries accounted for approximately a quarter of the total tax revenue on average (OECD 2023a). In this context, the structure of the income tax rate plays an essential role in determining economic growth (Stoilova 2017) and the aggregate welfare costs of taxation in an economy (Musgrave 1990). However, countries’ governments choose notably different tax rate structure strategies to collect personal income tax. For example, some Northern and Central European countries use a highly progressive tax system that taxes high incomes more heavily than low incomes (PWC 2023). In contrast, especially in postsocialist countries, taxing all income at a single rate, which is known as a flat tax, is commonplace (Domonkos 2016). The literature uses tax culture as a potential reason for differences across countries (Alesina and Glaeser 2004). In our definition, “a country-specific tax culture is the entirety of all relevant formal and informal institutions connected with the national tax system and its practical execution, which are historically embedded within the country’s culture, including the dependencies and ties resulting from their ongoing interaction” (Nerré 2001, p. 289). Thus, progressing globalization may lead to a growing population of individuals from not only diverse economic and social backgrounds but also distinct tax cultures.

Based on this consideration, this paper aims to shed light on the relationship between tax culture and preferences for tax rate structures. Our first research question addresses the general impact of tax culture on personal income tax rate structure preferences. In this context, we investigate whether migrants bring the tax culture of their country of origin to the destination country. Our second research question focuses on whether there is an assimilation of individuals with a second-generation migration background with the host tax culture. In other words, we examine whether second-generation migrants align their tax rate structure preferences with those of the host society.

We use a novel online vignette study to investigate these questions. Our sample consists of both migrants and non-migrants who are currently living in Germany. In the study, participants were instructed to envision themselves residing in a hypothetical German region. We then divided the fictitious population in this region into two groups: financially weak individuals and financially strong individuals. The participants’ task was to determine the average income tax rates of the financially weak and financially strong groups. We measure tax rate structure preferences using tax progressivity, which we calculate based on the difference between the tax rates of those two groups. To answer the questions of this paper, we examine different subsamples in the statistical analysis. To address the first research question, in the first step, we contrast non-migrants and migrants. In the second step, we distinguish participants with a migration background according to whether they originated from a country that levied income tax with a flat tax or not.1We define a flat tax country as a country that employed a flat tax rate to levy personal income tax for at least one year within the past 30 years. Table A1 in Appendix A shows all countries of origin of our subjects that we classify as flat tax countries. To answer the second research question, we divide participants with a migration background by generational status. In this regard, we contrast first-generation and second-generation migrants.

The German context of this study is appropriate for two reasons. First, Germany is the most frequently selected destination country for migrants in Europe and the second most important destination country worldwide behind the United States of America (McAuliffe and Triandafyllidou 2021). According to the German Federal Ministry of the Interior (BMI) and the Federal Office for Migration and Refugees (BAMF) more than every fourth inhabitant in Germany has a migration background (BMI/BAMF 2023). Thus, this study provides insight into how the tax rate structure preferences of a country are strongly affected by the impact of migration. Second, the German income tax rate is more progressive than the income tax rates of the ten most important countries of origin for persons with a migration background.2Table A2 in Appendix A provides a detailed comparison between the progressivity in income tax rates between Germany and its ten most important migration countries. Moreover, four of Germany’s most important migration countries (Bosnia and Herzegovina, Kazakhstan, Romania, and Russia) can be classified as flat tax countries, i.e. they use or have used a flat tax to levy income tax. The diversity of the population in Germany thus provides the opportunity for conducting a comprehensive study on the connection between tax culture and tax structure preferences.

Our results provide strong evidence for the general impact of tax culture on an individual’s tax rate structure preferences. Individuals with a migration background prefer a less-progressive income tax rate structure than those without a migration background. Moreover, migrants from countries with flat tax systems tend to prefer lower levels of income tax progressivity than migrants from countries with progressive tax systems. Concerning our second research question, our findings indicate that individuals with a migration background assimilate with the host society’s tax culture by the second generation. Consequently, first-generation migrants prefer a less-progressive income tax rate structure than second-generation migrants. However, our results also reveal that second-generation migrants still prefer a less progressive income tax rate than non-migrants.

With this paper, we contribute to the general literature that considers the perspective of migrants when examining the impact of societal heterogeneity on tax-related issues. While most of the prior literature has focused on the preferences of non-migrants, much less attention has been given to the preferences of people with migration backgrounds. Moreover, we contribute to the literature that employs the so-called epidemiological approach to investigate tax culture, i.e. using a migrant sample. While previous literature has relied on survey data, we create controlled settings with the help of a vignette approach. Moreover, this paper explains how the tax culture of individuals with a migration background is influenced by both the country-of-origin tax rate structure and the generational transition from the first to the second generation of migrants. These findings are relevant considering the increasing heterogeneity in modern societies and the growing influence of migrants on politics.

The remainder of this study is organized as follows. Section 2 uses theories and prior research findings for hypothesis formation. In Section 3, we provide an overview of the design and execution of the vignette study. Additionally, we detail the participants’ tasks and clarify the crucial variables considered for the statistical data analysis. Section 4 presents the results. Section 5 discusses the findings and provides an outlook for future research.

## 2 Literature and Hypothesis Development

For an extended period of time, empirical and experimental economic research has neglected the influence of culture on individual decisions. In particular, there needs to be a suitable approach for isolating and measuring potential cultural impacts. However, over the past 20 years, the number of related studies has increased substantially (Fernández 2011).3A literature review by Bisin and Verdier (2023) shows that the theoretical literature on cultural transmission has also evolved in recent years. Thus, the extant literature offers numerous hints and potential methodologies. In this context, according to prior work, researchers should consider two aspects. First, when conducting statistical analyses, importantly, not only potential cultural influences but also various determinants drive individual decisions. Second, it is vital to choose an approach that guarantees the differentiation of cultural factors from the impact of the economic and institutional environment in which the decision-making process takes place.

From an economic perspective, the model-theoretical framework of Meltzer and Richard (1981) provides the starting point for identifying factors influencing individual preferences for tax rate structures. In this model, individual preferences are determined exclusively by monetary self-interest. In the context of the tax rate structure, this relationship implies that those who (do not) benefit from redistribution prefer a higher (lower) degree of tax rate progressivity. Indeed, the literature shows strong evidence for the influence of self-interest on the preferred tax rate structure (Agranov and Palfrey 2015; Durante, Putterman, and Van Der Weele 2014; Esarey, Salmon, and Barrilleaux 2012; Hite and Roberts 1991; Klor and Shayo 2010). However, measuring and thus controlling for self-interest in statistical analysis poses challenges for researchers. Self-interest cannot be measured directly; thus, researchers must rely on the socioeconomic status of an individual, e.g. income or the level of education (Luttmer and Singhal 2011; Reeskens and Van Oorschot 2015). In addition to these objective measures, studies rarely consider subjective perceptions, such as perceived economic well-being, to capture self-interest (Fong 2001). Capturing self-interest with those imperfect proxies could result in measurement error. In contrast, the design of our vignette study allows us to ensure the systematic exclusion of monetary self-interest in the decisions of our participants.

In addition to self-interest, several empirical and experimental studies have identified numerous determinants that potentially drive individual tax rate structure preferences. Hennighausen and Heinemann (2015) found that individuals are more likely to prefer a progressive tax rate structure when they perceive the causes of inequality as unfair. Moreover, according to Ackert, Martinez-Vazquez, and Rider (2007), inequality-averse individuals prefer greater tax rate progressivity. Ballard-Rosa, Martin, and Scheve (2017) used a survey experiment to identify a significant influence of political orientation on tax progressivity preferences in the United States. Furthermore, Barnes (2015) showed that trust in government is negatively related to tax rate progressivity preferences. We will consider the results of these studies by controlling for the influence of these potential determinants in the statistical analysis.

The literature has used several approaches to measure cultural factors in economic decision-making. For example, researchers have examined cultural factors through historical case studies (Alesina and Fuchs-Schündeln 2007; Bondar and Fuchs-Schündeln 2023; Fisman and Miguel 2007; Torgler 2003). Other researchers have contrasted individual preferences across countries using empirical data (Alm and Torgler 2006; Cummings et al. 2004) or experimental studies (Henrich 2000; Kachelmeier and Shehata 1992; Roth et al. 1991). However, these approaches do not allow us to clearly differentiate between cultural factors and the economic and institutional context, as decision-making occurs across diverse settings (e.g. centuries or countries). For instance, Domonkos (2016) investigated tax rate structure preferences in Central and Eastern European countries and found a positive link between a country’s overall economic development and the average preference for a progressive income tax rate. To address this challenge and isolate cultural factors, we employ the “epidemiological approach”, which establishes a uniform setting by examining the behavior of migrants (Fernández 2008). Thus, our strategy borrows from a widely used approach in the literature, i.e. studying the economic impact of culture (Fernández 2011). Hedegaard (2023) identified two possible types of comparisons enabled by the use of migrants in this approach. The first is comparing the preferences of migrants in the destination country with the preferences of the host society in the migrants’ country of origin (Algan and Cahuc 2010; Antecol 2000; Chabé-Ferret 2019). Second, the influence of cultural factors can be isolated by contrasting either different groups of migrants within a destination country (Carroll, Rhee, and Rhee 1994; Giuliano 2007) or migrants and non-migrants (Breidahl and Larsen 2016; Dinesen and Hooghe 2010; Reeskens and Van Oorschot 2015). Our research strategy is grounded in the second type of comparison and thus ensures that decisions are made in an identical institutional and economic environment.

In the field of tax research, two studies have examined the relationship between tax culture and the redistribution of tax revenues using a migrant sample. First, Alesina and Giuliano (2011) used Word Values Survey data to address the fundamental question of whether migrants’ preferences for redistribution are shaped by their country-of-origin preferences. For this purpose, the researchers contrasted the preferences of migrants in the United States with the preferences of migrants from their countries of origin. The results showed a strong correlation between cultural factors and preferences for the redistribution of tax revenues. Second, Luttmer and Singhal (2011) used first- and second-generation migrants to examine the influence of culture on redistribution preferences in 32 European destination countries. The researchers used a question from the European Social Survey to contrast migrants’ preferences for redistribution with the average preferences of their country of origin. As a result, this study also showed the influence of tax culture. This outcome remained unchanged when various individual factors, such as income, education level, employment situation, and host country dynamics, were considered. A recent study by Hedegaard (2023) pursued a similar goal as that of our study by examining the general influence of tax culture on tax rate structure preferences. The researcher drew on the International Social Survey Program and Migrants’ Attitudes toward Welfare datasets to measure the influence of culture by contrasting migrants’ preferences with the average preferences of the country of origin. However, the survey items do not explicitly ask about tax rate progressivity preferences. For this reason, the dependent variable was calculated by the difference between attitudes toward high-income taxation and attitudes toward average middle- and low-income taxation. Hedegaard (2023) showed that migrants’ preferences for tax progressivity are shaped by the tax culture of their country of origin. While the studies above adopted an empirical approach using data analysis, our research examines the correlation between tax culture and tax rate structure preferences within the environment of a vignette study. In contrast to prior investigations, our research design ensures controlled conditions.

Our first primary research question addresses the general impact of tax culture on tax rate structure preferences. We use tax rate progressivity to measure tax rate structure preferences. Our hypothesis is rooted in the framework of policy-feedback theory, which posits that current policies influence the interests and beliefs of the public, subsequently impacting policy outcomes (Burstein 2003; Campbell 2012). Thus, current policy defines “normalcy” within a society, that is accepted as “the normal state of affairs”, and what is exceptional or even impermissible (Svallfors 2003). From the angle of prospect theory tax schemes participants have experienced serve as an anchor when asked to think of one’s preferences for tax progression (Tversky and Kahneman 1974). Consequently, we presume that the inclinations of individuals within a specific country concerning the progressivity of tax rates are already manifested in the existing income tax rate.4See also Hedegaard (2023), who deduced from the definition of tax culture that a country’s tax culture could be reflected in its policy orientation.

Thus, if we detect a difference between migrants and non-migrants or between subgroups of migrants in our vignette study, we can attribute those differences to different tax cultures. A comparison of income tax rate progressivity between Germany and its ten most important migration countries shows that Germany has the most progressive income tax rate, ranging from zero to 45 % (BMI/BAMF 2023; PWC 2023).5See Table A2 in Appendix A for an overview. Hence, we hypothesize that in our study, participants without a migration background will have a greater preference for a progressive tax rate than participants with a migration background. Furthermore, four of Germany’s most important migration countries (Bosnia and Herzegovina, Kazakhstan, Romania, Russia) can be classified as flat tax countries. Tax rate progressivity in flat tax countries is lower than that in nonflat tax countries. For this reason, we expect to find different tax rate structure preferences among our migrant participants based on a flat tax country migration background. Hypotheses 1, 2a, and 2b therefore state the following:

**Hypothesis 1:**

Participants with a migration background will prefer a less-progressive tax rate than participants without a migration background.

**Hypothesis 2a:**

Participants with a flat tax country migration background will prefer a less-progressive tax rate than nonflat tax country participants with a migration background.

**Hypothesis 2b:**

Participants with a nonflat tax country migration background will prefer a less-progressive tax rate than participants without a migration background.

Our second research question focuses on whether there is a tax cultural assimilation of individuals with a migration background over time. There are two theories in the literature that allow for prediction. First, researchers regularly rely on acculturation models (Berry 1997; Gordon 1964) to form hypotheses regarding the different behaviors of first- and second-generation migrants. On the one hand, according to Gordon’s (1964) unidimensional acculturation model, adaptation to the majority society cannot take place without simultaneously abandoning the culture of the country of origin. On the other hand, Berry’s (1997) bidimensional acculturation model treats the culture of origin and the culture of majority society as separate and distinct entities. According to Berry’s 2 × 2 matrix, an individual can choose between four different categories: assimilation (adaptation to the majority culture), integration (attachment to both cultures), segregation (reduction to the culture of origin) and marginalization (rejection of both cultures). In both models of acculturation, the strategy of assimilation implies an adaptation to the tax culture of the majority society.6In Berry’s (1997) model, an integration strategy could also lead to adaptation to the destination country’s culture. The indicators regularly examined within assimilation research are socioeconomic status or mobility, social relations, cultural beliefs, and political incorporation (Bloemraad, Korteweg, and Yurdakul 2008). Previous studies have provided evidence that politically, socially (Luttmer and Singhal 2011), and economically (Gereke, Schaub, and Baldassarri 2022) embedded migrants have similar preferences to non-migrants in the host society.

A second plausible approach used to explain differences in behavior between first- and second-generation migrants is provided by Durkheim’s (1951) social integration theory. According to the theory, individuals who are strongly socialized in a social group are assumed to be more likely to comply with the values, norms, and attitudes of this group (Van Tubergen 2007). Durkheim’s (1951) theoretical framework is widely used in migration research, e.g. in the context of the acculturation of secularization (Van Der Bracht, Van De Putte, and Verhaeghe 2013) or national pride (Reeskens and Wright 2014). Reeskens and Van Oorschot (2015) found evidence for this theory in the context of preferences for the redistribution of tax revenues.

A common feature of both theories is that they contain a temporary component. Lorenzo-Hernández (1998) argued that the process of assimilation regularly begins with the second generation. A systematic literature review by Drouhot and Nee (2019) revealed that in terms of socioeconomic attainment, social relations and cultural beliefs, second-generation migrants overall adapt better to the host society than first-generation migrants. However, researchers have shown that social factors such as religious differences or a lack of citizenship inhibit assimilation. Consequently, some groups of migrants cannot assimilate even in the second generation. Moreover, Guiso, Sapienza, and Zingales (2006) argued that distinct values, norms and attitudes are deeply rooted in individuals differently and therefore require different periods for assimilation.

Drawing from these findings, we argue that as migrants assimilate, they align with the tax rate structure preferences of non-migrants. Moreover, we assume that second-generation migrants are assimilated to a greater degree by the host society than first-generation migrants. Therefore, second-generation migrants are expected to have more non-migrant preferences than first-generation migrants. According to Hypothesis 1, we expect that migrants will prefer a less-progressive tax rate than non-migrants. Accordingly, we hypothesize that first-generation migrants prefer a less-progressive tax rate than second-generation migrants. However, we anticipate that not all second-generation migrants will have assimilated the host society’s tax culture. On the one hand, some second-generation migrants may have opted for an acculturation strategy that does not adopt the tax culture of non-migrants. On the other hand, adaptation could have been hindered by various complicating factors or time constraints. For this reason, we assume that second-generation migrants will prefer a less-progressive tax rate structure than non-migrants. Hypotheses 3a and 3b therefore state the following:

**Hypothesis 3a:**

Participants with a first-generation migration background will prefer a less-progressive tax rate than participants with a second-generation migration background.

**Hypothesis 3b:**

Participants with a second-generation migration background will prefer a less-progressive tax rate than participants without a migration background.

## 3 Design and Setup of the Vignette Study

### 3.1 Overview

Our research is based on an extensive vignette study that investigates the link between taxation and migration in a multifaceted way.7This experimental vignette study is part of the Fiscal Citizenship Project, which is a multi-disciplinary effort investigating the connection between migration and taxation. This vignette study supports the project by investigating and contrasting different tax collection and tax usage preferences among migrants and non-migrants. For further information, see: https://www.uni-wuerzburg.de/en/research/the-fiscal-citizenship-project. This paper addresses one specific question. Notably, not all facets and content within the vignette study bear relevance to the scope of this paper. For this reason, in the following, we describe the design and setup of the vignette study only insofar as it is relevant to answering the research question of this paper.

**Figure 1:**
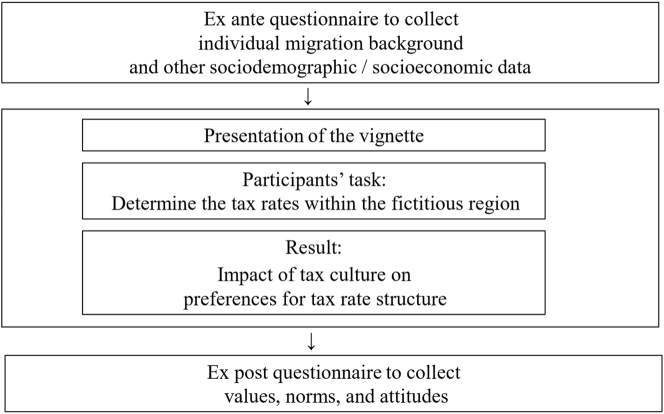
Overview of the vignette study.

First, this chapter provides an overview before describing the relevant components and the procedure of our research method in detail. This paper aims to investigate the relationship between tax culture and tax rate structure preferences. For this purpose, we use a novel vignette study. Combining survey and experimental research methods allows us to compensate for the shortcomings of each approach and harness their strengths (Atzmüller and Steiner 2010; Hox, Kreft, and Hermkens 1991). To isolate tax culture, our sample comprises participants without a migration background and with a migration background. To facilitate a comparison between migrants and non-migrants in the subsequent analysis, participants had to adhere to their real-life migration status in the controlled environment of the vignette study. Hence, it was crucial to gather information about a participant’s specific migration background through an ex ante questionnaire before introducing this information to the vignette. After the participants had read the vignette, they were confronted with a decision-making situation. Their task was to determine the average income tax rate for both the financially weak and strong groups in a fictional region of Germany. Finally, an ex post questionnaire was used to collect the participants’ values, norms, and attitudes. Figure 1 illustrates this overview.

### 3.2 Vignette Study

This study utilizes an online vignette approach. This methodology combines a traditional questionnaire with a vignette experiment. While the above literature review demonstrates that investigating survey data is a commonly chosen methodology in tax culture research, there is a limited body of experimental work in this field (Fernández 2008).

Our approach compensates for the typical shortcomings of laboratory experiments (Fernández 2011). First, it overcomes the issue of small and often student-centered experimental samples by utilizing an online design, allowing us to include a larger and more diverse population sample (Auspurg and Hinz 2015; Mutz 2011). Second, it addresses the challenge of unaccounted individual participant attributes by incorporating a questionnaire component, which provides the opportunity to collect a comprehensive range of control variables (Aguinis and Bradley 2014). Furthermore, utilizing a vignette approach offers additional advantages in comparison to traditional survey data. On the one hand, the presentation of hypothetical scenarios related to sensitive topics such as migration (Lane 2016) helps to avoid response biases and the social desirability effect (Auspurg and Hinz 2015). On the other hand, a vignette portrays a problem or scenario within a multifaceted context. Therefore, when contrasted with a conventional survey, a vignette offers a more realistic and less abstract means of conveying information (Steiner, Atzmüller, and Su 2016). Consequently, a vignette study is better suited for identifying policy attitudes than a traditional survey (Mutz 2011; Petersen et al. 2011).

### 3.3 Description of the Vignette

The structure of the vignettes was consistent for all participants. The language of implementation was German, and straightforward terminology was employed for all explanations. In addition, pictures clarified the essential content of the vignette.8An example of a vignette is provided in Appendix B.1 (Supplementary Material). We used a between-subject design. The presented vignette asked our participants to envision themselves living in an imaginary region within Germany inhabited by 60,000 individuals. The participants were directed to consider that half of these inhabitants (30,000 individuals) had a migration background, while the other half did not. Moreover, this hypothetical society was equally divided between financially weak and financially strong inhabitants. In other words, within this vignette were two groups of 30,000 inhabitants, namely, one financially strong and the other financially weak. Within this vignette, we manipulated two dimensions relevant to our study.9In addition, we also vary a third dimension. However, this Financial Status Ingroup dimension is not relevant to the research question of this paper and is therefore not explained. However, this variation could lead to group interests among participants that influence the decision-making process. Our experimental design mitigates potential bias by ensuring participants were equally exposed to financially strong and weak ingroups. First, we varied the individual migration status. However, each participant’s migration status in this fictitious German region needed to mirror his or her real-life migration status. To achieve this alignment, we obtained the necessary information for classification from the ex ante questionnaire. In the case where a respondent stated that he or she did (not) have a migration background in Germany, he or she was assumed (not) to have a migration background in our invented German region. Second, we randomly assigned participants to either the financially strong or financially weak status. In other words, this dimension was used to assign the participants to either the financially weak or financially strong group within the (fictitious) society. The assignment of an individual financial status allows the participants themselves to be part of the region and thus increase their identification with the region. Participants were equally distributed between financially weak and strong groups to prevent potential bias. As this dimension is relative and highly subjective, it varied independently of the net household income indicated in the ex ante questionnaire and was exclusively based on the random principle.

### 3.4 Participants’ Task and Measurement of Tax Progressivity

Once a participant had read and comprehended the vignette, we initiated the study task with the goal of revealing our participants’ tax progressivity preferences.10After the participants had read the vignette, we asked them comprehension questions about the main content of the vignette. The comprehension questions can be found in Appendix B.2 (Supplementary Material). For two reasons, tax rate structure preferences are an effective method for measuring tax culture. First, besides tax revenue usage, the examined tax collection is a fundamental component of taxes. In this context, various tax schedules can raise the same amount of tax revenue. The degree of progressivity of the tax rate thus represents a central political decision that significantly influences the distribution of the tax burden among all members of the population and the redistributive character of a state (Ballard-Rosa, Martin, and Scheve 2017). Secondly, setting tax rates ensures a simple and understandable design for participants. Richer vignettes of tax culture would have borne the risk of not being understood by participants and would have made interpretation over-complex. In contrast to other studies using survey items, we chose to utilize a decision-making scenario for this purpose.11See Appendix B.3 (Supplementary Material) for the instructions of the decision-making situation. The participants’ task was to manipulate the average income tax rates within the invented region in Germany. In particular, these adjustments spanned the financially strong and financially weak groups. In each case, the initial tax rate values were set exogenously and identically for each participant: the average income tax rate for the financially strong group was 25 %, while it was 10 % for the financially weak group. A participant could specify an (integer) average tax rate between 0 % and 100 % for both financial groups. Accordingly, to conceal the purpose of the study, this decision-making situation did not directly target participants’ tax rate progressivity preferences. However, we calculated individual preferences for progressivity based on the difference between the average tax rate of the financially strong and financially weak groups. Thus, our dependent variable “Tax_Progressivity” was determined as follows:

Tax_Progressivity=Tax_Rate_Strong_Group−Tax_Rate_Weak_Group

For example, if a participant left the average tax rate of both groups unchanged, the tax rate progressivity remained at the initial level of 15 percentage points (25 %–10 %). On the other hand, if the average tax rate of the financially strong group increased by five percentage points and the tax rate of the financially weak group decreased by five percentage points, the tax progressivity would be 25 percentage points (30 %–5 %).

**Figure 2:**
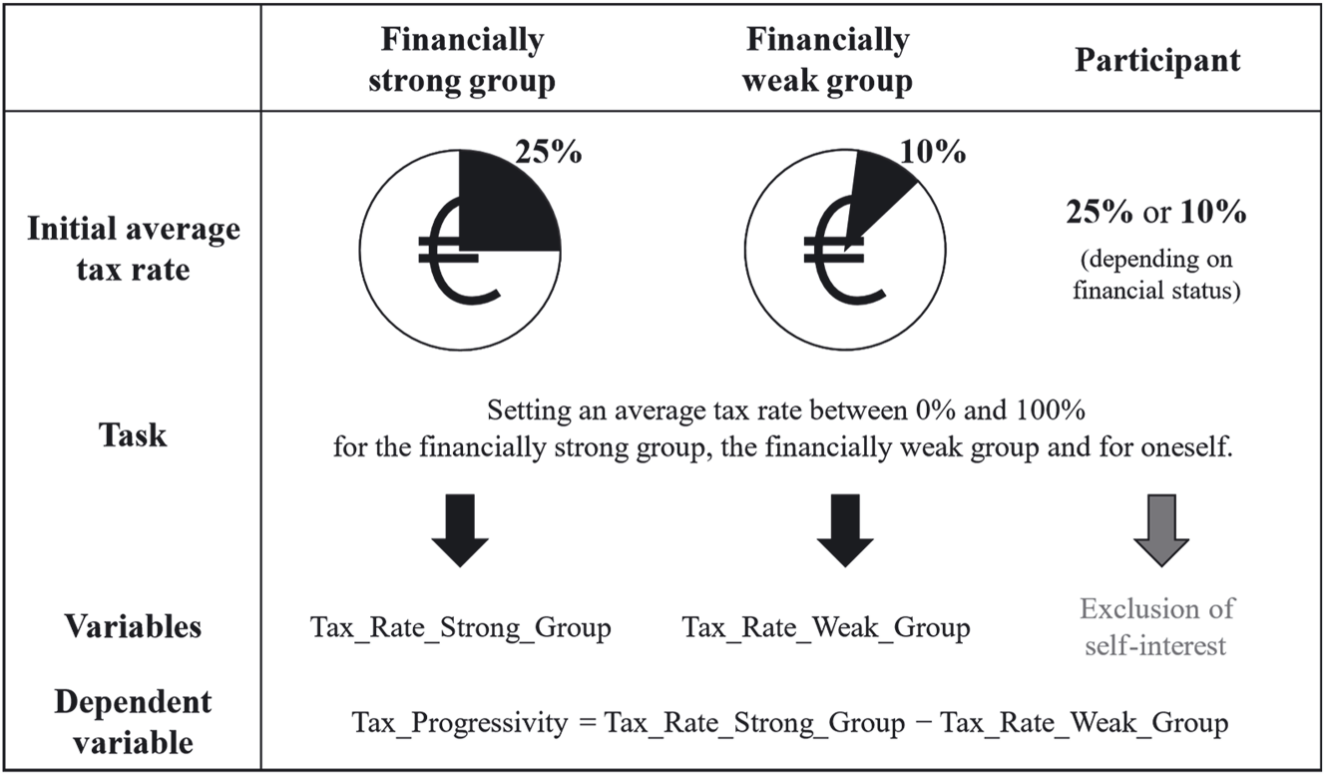
Formation of the dependent variable. **Note.** Figure 2 summarizes the process from the participants’ task to the formation of the dependent variable. The task of the participants is to adjust the tax rate of both the financially weak group and the financially strong group according to their personal preferences. The initial tax rate for the financially strong group was 25 %, while it was 10 % for the financially weak group. The variable Tax_Rate_Strong_Group captures the adjusted tax rate of the financially strong group and the variable Tax_Rate_Weak_Group captures the adjusted tax rate of the financially weak group. The dependent variable of the statistical data analysis Tax_Progressivity is the difference of the variables Tax_Rate_Strong_Group and Tax_Rate_Weak_Group. To exclude the impact of self-interest, participants set their personal tax rate independently of the tax rates of the groups.

Furthermore, we designed the task scenario in a way that eliminated the impact of a participant’s self-interest on the tax rates within the fictitious society groups. For this purpose, we allowed the participants to manipulate their personal average income tax rate independently of the tax rate of their financial group. The initial level of the personal tax rate depended on a participant’s assigned individual financial status; i.e. the tax rate was either 25 % or 10 %. For example, if the preferences of a personally financially strong participant were driven exclusively by monetary self-interest, this participant would not change the tax rate of the financially strong group but would reduce the personal average income tax rate from the initial level of 25 %–0 %. By excluding the impact of participants’ self-interest on the tax rate of the financial groups, we aimed to ensure that decisions made for the (fictional) society were more objective, neutral, and independent. This approach aimed to maintain tax rate comparability in the statistical analysis by deterring self-interest from influencing the tax rates of either group. Additionally, by eliminating self-interest from the calculation of the preferred tax rate progressivity for society, our results can be considered more policy relevant. Figure 2 outlines the process from the participants’ task to the formation of the dependent variable.

Another measure taken to exclude the influence of self-interest is that the participants’ decisions were not monetarily incentivized. The monetary incentivization of decisions in the experimental methodology can be used to gain more control over the participants’ preferences. However, the questions in this study require the exclusion of the impact of self-interest. For this reason, incentivizing individual decisions would have been counterproductive. Instead, the participants in this study receive a lump sum remuneration which does not depend on any decision they have taken during the experiment. Instead, the remuneration is based on the German minimum wage and depends on the length of time each participant spends on the study.

### 3.5 Questionnaires and Variables

We asked the participants to complete two questionnaires.12The ex ante and ex post questionnaires can be found in Appendix B.4 (Supplementary Material). The ex ante questionnaire asked for the country of birth of both the participant and his or her parents. The country of birth could be selected from a list of all countries in the world. This information allowed us to make various classifications of our subjects for testing our hypotheses.

In the first step, we determined the migration status of a respondent. For the classification, we followed the OECD definition, according to which a migration background exists if a person or at least one of his or her parent was not born in the country of destination (OECD/EU 2018). We used these data to assign a migration status to participants in the fictional region of our study that accurately mirrored their migration status in real life. In addition, migration status served as an independent variable in the statistical analysis, as Hypothesis 1 compares the tax structure preferences of participants with and without a migration background. To compare the groups, the dummy variable “MB” was used, which takes the value of 1 if a migration background is present and 0 otherwise. In addition, the dummy variable “NO_MB” was created, which takes the value of 1 if the participant and his or her parents were born in Germany and 0 otherwise.

Hypotheses 2a and 2b require differentiating participants with a migration background according to whether they have a migration background stemming from a flat tax country. According to our definition, a flat tax country is a country that employed a flat tax system to levy personal income tax for at least one year within the past 30 years.13Table A1 in Appendix A shows all countries of origin of our subjects that we classified as flat tax countries. Based on this, we certified a respondent has having a flat tax migration background if either the respondent or at least one of his or her parents was born in a flat tax country. For later analysis, we created the following two dummy variables, namely, the dummy variable “MB_FLAT”, which takes the value of 1 if a participant has a flat tax migration background and 0 otherwise, and the dummy variable “MB_NO_FLAT”, which takes the value of 1 if neither the migrant participant nor one of his or her parents was born in a flat tax country and 0 otherwise.

Finally, we needed to identify the generational status of our migrant participants to test Hypotheses 3a and 3b. A participant was classified as belonging to the first generation of migrants, regardless of his or her parents’ birthplace, if he or she was not born in Germany. A participant with a migration background was seen as belonging to the second generation of migrants if he or she was born in Germany and at least one of his or her parents was not born in Germany. Again, our statistical analysis accounted for generation status with the help of two dummy variables. On the one hand, the dummy variable “MB_1st_GEN” takes the value of 1 if a participant belongs to the first generation of migrants and 0 otherwise. On the other hand, the dummy variable “MB_2nd_GEN” takes the value of 1 if a participant belongs to the second generation of migrants and 0 otherwise.

In addition, the ex ante questionnaire collected further sociodemographic and socioeconomic information on the participants, which was used as a control variable in later analyses (age, gender, income, and education). In the ex post questionnaire, we asked for sensitive values, norms and attitudes of the participants. From these items, we included a selection as control variables in the statistical data analysis, which could influence preferences for tax rate progressivity (e.g. fairness beliefs about effort, attitudes toward income equalization, political orientation, and trust in government). An overview of all variables used in this study is provided in Table C1 of Appendix C (Supplementary Material).

### 3.6 Setup

The vignette study was carried out by the panel data provider Münster Research Institute14The Münster Research Institute (MRI), founded in 2003 and based in Münster, provides scientific services, particularly in obtaining social and economic empirical data. For further information see: https://muenster-research.de/. and took place from September 20, 2022, to October 5, 2022. The online opt-in sample could complete the study via a personal computer, tablet or smartphone. Unlike a traditional laboratory experiment with a student sample, our online design improved participant accessibility. This allowed us to efficiently recruit a sufficient number and diverse range of participants, especially those with a migration background, to answer our research questions adequately. Quotas were used to achieve an appropriate ratio of participants without a migration background to those with a migration background. After data cleaning, the analyzed dataset for this study comprised a total of 2,422 participants.15A total of 2,796 participants completed the study. Nevertheless, we eliminated participants whose survey completion time was below 50 % of the mean to ensure high data quality standards. Moreover, we excluded participants with an Iraqi (5 participants) or Tajik (1 participant) migration background from the analyses related to the impact of a flat tax migration background unless they possessed a flat tax migration background from another country. This exclusion was necessary because of inconsistent information concerning the income tax rate structure in these countries. Within this sample, 840 participants had no migration background, while 1,582 participants had a migration background. Table 1 provides an overview of the sociodemographic data for the total sample and the subsamples of the analysis. Detailed summary statistics of the control variables for the subsamples and the total sample presented in Table 1 can be found in Tables D1 and D2 of Appendix D (Supplementary Material).

**Table 1:** Participants demographics’.

(Sub) sample	No MB	MB	MB FLAT	MB NO FLAT	MB 1st GEN	MB 2nd GEN	Total
*N*	840	1,582	424	1,152	900	682	2,422
Age (median)	39	32	32	32	34	28	34
Female	48.45 %	61.00 %	69.81 %	57.90 %	58.89 %	63.78 %	56.65 %
Income (median in euros)	2,000.01–2,600.00	2,000.01–2,600.00	2,000.01–2,600.00	2,000.01–2,600.00	2,000.01–2,600.00	2,000.01–2,600.00	2,000.01–2,600.00
Academic	32.74 %	35.97 %	37.97 %	35.42 %	40.33 %	30.21 %	34.85 %
Employed	75.48 %	73.14 %	72.41 %	73.44 %	74.33 %	71.55 %	73.95 %
Tax knowledge	2.83	2.90	2.88	2.91	2.89	2.92	2.88

**Note.** Table 1 shows descriptive statistics of the demographic characteristics of the participants. A distinction is made between different subsamples and the total sample. “Income” uses an ordinal scale measures the net household income in euros: (1) <900.00 (2) 900.00–1,299.99 (3) 1,300.00–1,499.99 (4) 1,500.00–1,999.99 (5) 2,000.00–2,599.99 (6) 2,600.00–3,599.99 (7) 3,600.00–4,999.99 (8) ≥5,000.00. “Academic” indicates whether a participant has achieved an academic degree within Germany or an equivalent qualification from abroad. “Employed” includes full-time, part-time, or self-employed participants. “Tax Knowledge” is measured with an ordinal scale (1 = “None at all” – 5 = “Above average”).

## 4 Results

Our results are presented as follows. First, in Subsection 4.1, we address Hypotheses 1, 2a, and 2b, exploring the general impact of tax culture on tax rate structure preferences. In Subsection 4.2, we address Hypotheses 3a and 3b. In this subsection, we introduce a temporal dimension to the analysis by distinguishing between first- and second-generation migration backgrounds. In Subsection 4.3, the findings on the influence of cultural factors over time are expanded by introducing and analyzing further subgroupings of participants with a migration background. In Subsection 4.4, we perform robustness checks. To prove the statistical significance of the results, we use multiple ordinary least square regression (OLS) with robust standard errors, considering a rich set of control variables. The dependent variable of each regression analysis is the metric variable Tax_Progressivity. The results presented in the figures of this chapter show the average of the variable Tax_Progressivity.

### 4.1 Hypotheses 1, 2a, and 2b

**Figure 3:**
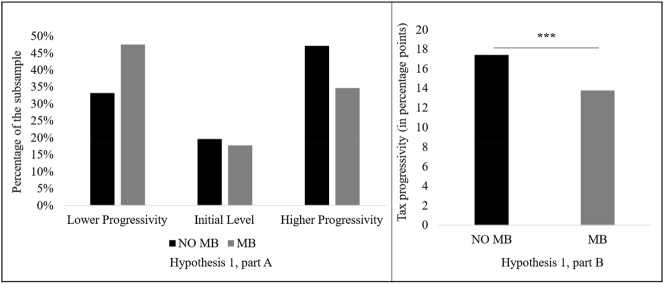
The preferences for tax rate progressivity by migration status. **Note.** Figure 3 compares the preferences for tax rate progressivity between NO MB participants and MB participants. Figure 3 (Hypothesis 1, part A) shows the distribution of preferences for lower progressivity, the initial level, and higher progressivity in percentages. Figure 3 (Hypothesis 1, part B) illustrates the average value of the metric variable Tax_Progressivity in percentage points.

To test Hypothesis 1, we compare the tax rate progressivity preferences of participants without a migration background (NO MB) and participants with a migration background (MB). We expect that MB participants will prefer a less-progressive tax rate than NO MB participants. Figure 3 (Hypothesis 1, part A) provides an overview of whether the tax rate progressivity has increased, decreased, or remained at the initial level of 15 percentage points due to the participants’ decisions. The figure distinguishes between NO MB participants and MB participants. The figure shows that the progressivity remains unchanged for approximately every fifth NO MB participant and 18 % of the MB participants. An increase in progressivity is preferred by 47 % of NO MB participants and 35 % of MB participants. In contrast, 48 % of MB participants prefer a decrease in progressivity, and 33 % of NO MB participants prefer a decrease in progressivity. Figure 3 (Hypothesis 1, part B) compares the mean values of participants’ preferred tax progressivity by migration status. It shows that the average tax rate progressivity for NO MB participants is 17.39 percentage points, while that of the MB participants is 13.74 percentage points.

Table 2 shows the results of the regression analysis. Regression Model 1 examines whether tax rate progressivity preferences differ between migration statuses. The reference group is the population of NO MB participants. The coefficient of the dummy variable MB measures the difference in the tax rate progressivity between the MB participants and the reference group. The model uses control variables. The regression analysis results show a negative sign for the coefficient of the variable MB at a statistically significant level. This result indicates that MB participants prefer a less-progressive tax rate than NO MB participants. Thus, the analysis supports Hypothesis 1.

**Table 2:** Hypotheses – OLS results.

Subsamples	Hypothesis 1	Hypothesis 2a	Hypothesis 2b	Hypothesis 3a	Hypothesis 3b
	Model 1	Model 2	Model 3	Model 4	Model 5
Reference	NO_MB; MB	NO_MB; MB_NO_FLAT;		NO_MB; MB_1st_GEN;	
group		MB_FLAT		MB_2nd_GEN	
	NO_MB	MB_NO_FLAT	NO_MB	MB_2nd_GEN	NO_MB
Constant	14.448***	11.972***	14.437***	12.502***	13.781***
	(1.619)	(1.581)	(1.621)	(1.584)	(1.618)
MB	−2.768***				
	(0.505)				
NO_MB		2.466***		1.279**	
		(0.536)		(0.633)	
MB_FLAT		−1.116*	−3.582***		
		(0.638)	(0.683)		
MB_NO_FLAT			−2.466***		
			(0.536)		
MB_1st_GEN				−2.463***	−3.742***
				(0.603)	(0.549)
MB_2nd_GEN					−1.279**
					(0.633)
Controls	Yes	Yes	Yes	Yes	Yes
*N*	2,411	2,406	2,406	2,411	2,411
Adjusted *R*^2^	0.068	0.069	0.069	0.075	0.075

***p ≤ 0.01; **p ≤ 0.05; *p ≤ 0.10. **Note.** Table 2 reports OLS results (robust standard errors in parentheses). In each model the dependent variable is the metric variable Tax_Progressivity. Model 1 tests Hypothesis 1. The sample is divided by migration status. NO MB participants are the reference group. Model 2 tests Hypothesis 2a and Model 3 tests Hypothesis 2b. The sample is divided by migration status and MB participants are sorted by the classification characteristic of flat tax migration background. In Model 2 MB NO FLAT participants are the reference group and in Model 3 NO MB participants are the reference group. Model 4 tests Hypothesis 3a and Model 5 tests Hypothesis 3b. The sample is divided by migration status and MB participants are sorted by the classification characteristic of generational status. In Model 4 MB 2nd GEN participants are the reference group and in Model 5 NO MB participants are the reference group. Controls are not reported here but can be found in Appendix E, Table E1 (Supplementary Material). All variables are defined as described in Appendix C, Table C1 (Supplementary Material).

**Figure 4:**
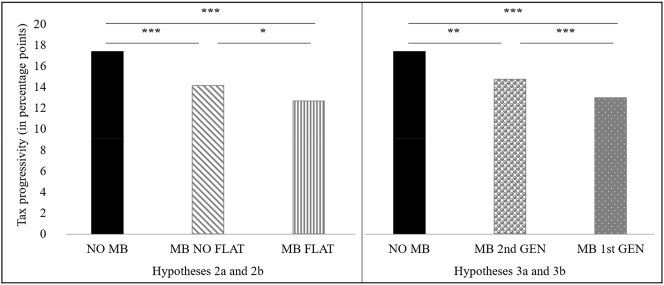
The preferences for tax rate progressivity by migration status and subgroups of migrants. **Note.** Figure 4 compares the preferences for tax rate progressivity between NO MB participants and subgroups of MB participants in percentage points. In Figure 4 (Hypotheses 2a and 2b), MB participants are sorted by the classification characteristic of a flat tax migration background. Accordingly, the MB participants are divided into MB NO FLAT participants and MB FLAT participants. In Figure 4 (Hypotheses 3a and 3b), MB participants are sorted by the classification characteristic of generational status. Accordingly, the MB participants are divided into MB 2nd GEN participants and MB 1st GEN participants.

In the following, we test Hypotheses 2a and 2b. These hypotheses suggest distinct tax cultures among participants with migration backgrounds, driven by differences in tax rate structures in their countries of origin. To test the hypotheses, we differentiate within the MB participants between participants with a flat tax migration background (MB FLAT) and participants without a flat tax migration background (MB NO FLAT). We expect MB FLAT participants to prefer a less progressive income tax rate than MB NO FLAT participants (Hypothesis 2a). In addition, we expect MB NO FLAT participants to choose a less progressive income tax rate than NO MB participants (Hypothesis 2b). Figure 4 (Hypotheses 2a and 2b) provides an overview of the average tax rate progressivity within the subgroupings of our sample. While MB NO FLAT participants have an average tax rate progressivity of 14.15 percentage points, the extent of the tax rate progressivity for MB FLAT participants is 12.67 percentage points. NO MB participants choose a more progressive income tax rate than MB NO FLAT participants, making it the most progressive tax rate choice in this comparison.

Table 2 shows whether the differences are statistically significant. The crucial independent variables are the dummy variables that differentiate the sample by migration status and flat tax migration background (MB_FLAT, MB_NO_FLAT, NO_MB). On the one hand, to test Hypothesis 2a in Regression Model 2, MB NO FLAT participants comprise the reference group. On the other hand, to test Hypothesis 2b in Regression Model 3, NO MB participants serve as the reference group. The coefficient of the dummy variable in the regression model consistently measures the difference from the reference value. Both models include control variables. The results of Model 2 confirm a statistically significant difference in preferences for progressivity regarding the personal income tax rate between MB NO FLAT participants and MB FLAT participants. The coefficient on the dummy variable MB_FLAT is negative and statistically significant at the 10 % level. This result provides support for Hypothesis 2a. Moreover, the income tax rate is significantly less progressive for MB NO FLAT participants than for NO MB participants (Model 3). This result is consistent with Hypothesis 2b. In summary, comparing subparticipants from countries with different tax rate structures leads to the following conclusion:

**Result 1:**

Participants with a migration background prefer a less progressive income tax rate than participants without a migration background. Migrants with a flat tax migration background prefer a less progressive income tax rate than migrants without a flat tax migration background.

### 4.2 Hypotheses 3a and 3b

Hypotheses 3a and 3b are tested to determine whether there is a tax cultural assimilation of people with a second-generation migration background with the host culture. For this purpose, we subdivide the MB participants in our sample according to their first-generation migration background (MB 1st GEN) and second-generation migration background (MB 2nd GEN). We expect MB 1st GEN participants to prefer a less progressive income tax rate than MB 2nd GEN participants (Hypothesis 3a). In addition, we expect MB 2nd GEN participants to prefer a less progressive income tax rate than NO MB participants (Hypothesis 3b). Figure 4 (Hypotheses 3a and 3b) provides an overview of the average progressivity in the income tax rate of the groups. Figure 4 shows that for MB 2nd GEN participants, the average level of progressivity is 14.75 percentage points, while for MB 1st GEN participants, the average level of tax progressivity is 12.97 percentage points. The most progressive income tax rate results are for NO MB participants.

Table 2 shows the OLS results. For this analysis, we use the dummy variables that distinguish participants in our sample by migration status and generational status (MB_1st_GEN, MB_2nd_GEN, NO_MB). To test Hypothesis 3a, MB 2nd GEN participants comprise the reference group in Model 4. In addition, for testing Hypothesis 3b in Model 5, NO MB participants serve as the reference group. Both models account for control variables. In Model 4, the coefficient of the dummy variable MB_1st_GEN is negatively pronounced at a statistically significant level. This result indicates that MB 1st GEN participants prefer a statistically significantly less-progressive tax rate than MB 2nd GEN participants. Thus, Hypothesis 3a is confirmed. Furthermore, Model 5 shows that the tax rate adopts a statistically significantly lower degree of progressivity for MB 2nd GEN participants than for NO MB participants. This result is consistent with Hypothesis 3b.

Overall, the distinction between first-generation and second-generation migrants allows us to conclude the following:

**Result 2:**

Participants with a first-generation migration background tend to prefer a less progressive income tax rate than participants with a second-generation migration background. Participants with a second-generation migration background prefer a less progressive income tax rate than participants without a migration background.

### 4.3 Further Analysis

This further analysis aims to provide more detailed insight into the relationship between tax culture and tax rate structure preferences. For this purpose, we jointly apply the classification characteristics of flat tax migration background and generational status, which were used separately in the previous subsections. Accordingly, the participants with a migration background are divided into four subgroups in this section: (1) participants with a flat tax migration background of the first generation (MB FLAT 1st GEN), (2) participants without a flat tax migration background of the first generation (MB NO FLAT 1st GEN), (3) participants with a flat tax migration background of the second generation (MB FLAT 2nd GEN) and (4) participants without a flat tax migration background of the second generation (MB NO FLAT 2nd GEN). In addition, NO MB participants are included in the analysis. Table 3 provides an overview of the subgroups of MB participants for further analysis.16A detailed summary statistics of control variables for the MB participant subgroups of further analysis can be found in Table D3 of Appendix D (Supplementary Material).

**Table 3:** Subgroups of MB participants subjected to further analysis.

		Flat tax migration background	
		Yes	No
Generational status	1^st^ Generation	MB FLAT 1st GEN	MB NO FLAT 1st GEN
	2^nd^ Generation	MB FLAT 2nd GEN	MB NO FLAT 2nd GEN

**Note.** Table 3 presents an overview of the subgroups among MB participants for further analysis. In this context, the classification characteristics of flat tax migration background and generation status are applied simultaneously. Consequently, participants with a migration background are divided into MB FLAT 1st GEN, MB NO FLAT 1st GEN, MB FLAT 2nd GEN, and MB NO FLAT 2nd GEN participants.

Two aspects of this further analysis are of particular relevance to our research questions. On the one hand, the analysis allows us to compare the tax rate structure preferences of first-generation migrants based on whether they have a flat tax migration background. Thus, we obtain a more detailed understanding of whether individuals with a migration background carry the tax culture of their country of origin to their destination country. On the other hand, this analysis allows us to compare the extent to which second-generation migrants adapt to the preferences of the host society depending on a flat tax migration background. Therefore, the analysis reveals additional insights into the tax cultural assimilation of migrants with the host society as a function of their country of origin.

**Figure 5:**
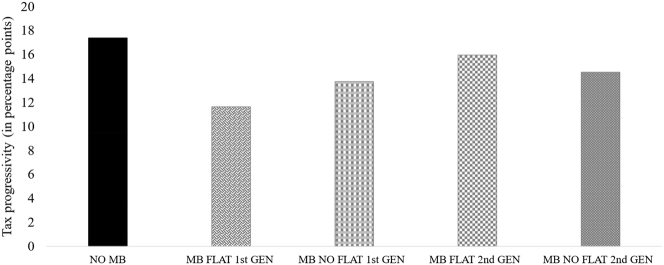
Further analysis of the preferences for tax rate progressivity. **Note.** Figure 5 compares the preferences for tax rate progressivity between NO MB participants and subgroups of the MB participants in percentage points. In this figure, MB participants are sorted by the classification characteristics of flat tax migration background and generational status simultaneously. Accordingly, the MB participants are divided into MB FLAT 1st GEN, MB NO FLAT 1st GEN, MB FLAT 2nd GEN, and MB NO FLAT 2nd GEN participants.

Figure 5 provides an overview of the average level of tax rate progressivity among the subgroups. First, the focus is on participants with a first-generation migration background. Second, we consider second-generation migrant participants. While the level of tax rate progressivity for MB FLAT 2nd GEN participants is 15.96 percentage points, the MB NO FLAT 2nd GEN participants exhibit a progressivity level of 14.54 percentage points. Thus, independent of a flat tax migration background, this result reveals an increase in average tax rate progressivity for second-generation migrants compared to first-generation migrants. The highest overall tax rate progressivity is observed among NO MB participants.

We use OLS regression analyses to test the observed differences for statistical significance. For this purpose, we form a dummy variable (MB_FLAT_1st_GEN, MB_NO_FLAT_1st_GEN, MB_FLAT_2nd_GEN, MB_NO_FLAT_2nd_GEN) for each subgroup within the MB participants (see Table 3). For example, the dummy variable MB_FLAT_1st_GEN takes the value of 1 if a participant has a first-generation and flat tax migration background and 0 otherwise. In addition, the dummy variable NO_MB is included in each regression model. To test all the differences for statistical significance, four regression analyses are conducted. The models follow an analogous procedure and differ only in the reference group. The dependent variable is the metric variable Tax_Progressivity. Each regression model includes control variables. The main results of the regression models are reported below.

Table 4 reports the OLS results. Model 1 uses MB FLAT 1st GEN participants as the reference group. Specifically, this model compares the preferences of first-generation migrants with and without flat tax migration backgrounds. The results indicate that MB FLAT 1st GEN participants tend to prefer a lower tax rate progressivity than that preferred by MB NO FLAT 1st GEN participants. Additionally, all other predictors in the subgroupings show statistical significance. Thus, this model suggests a greater preference for tax progressivity by MB FLAT 2nd GEN participants than by MB FLAT 1st GEN participants. In Model 2, the MB NO FLAT 1st GEN participants serve as the reference group. The focus of this model is on the comparison between the first and second generations of migrants without a flat tax migration background. In this context, the model shows that the MB NO FLAT 2nd GEN participants prefer a significantly greater tax rate progressivity than that preferred by the MB NO FLAT 1st GEN participants. Model 3 uses the MB FLAT 2nd GEN participants as the reference group. This model enables a comparison of the subgroups of second-generation migrants and allows a contrast with the host society. The results of this model show a statistically significant difference for neither the MB NO FLAT 2nd GEN participants nor the NO MB participants. Recall that MB FLAT 1st GEN participants were previously found have the lowest preference for our subgroupings for a progressive tax rate. This result makes it all the more remarkable that MB FLAT 2nd GEN participants are shown to have adapted to the preferences of the NO MB participants. Model 4 uses the MB NO FLAT 2nd GEN participants as the reference group. Analogous to Model 3, we use the model to observe differences between the reference group and the NO MB participants. The results reveal that MB NO FLAT 2nd GEN participants group prefer a lower tax rate progressivity than that preferred by NO MB participants. Thus, compared to those of the MB NO FLAT 1st GEN participants, the preferences for a progressive tax rate of MB NO FLAT 2nd GEN participants increase, but they still do not reach the level of the host society.

**Table 4:** Further analysis – OLS results.

Reference group	Model 1	Model 2	Model 3	Model 4
	MB_FLAT_1st_GEN	MB_NO_FLAT_1st_GEN	MB_FLAT_2nd_GEN	MB_NO_FLAT_2nd_GEN
Constant	9.201***	10.501***	13.598***	12.237***
	(1.690)	(1.635)	(1.999)	(1.583)
NO_MB	4.565***	3.265***	0.168	1.529**
	(0.737)	(0.611)	(1.282)	(0.667)
MB_FLAT_1st_GEN		−1.300*	−4.397***	−3.036***
		(0.759)	(1.380)	(0.782)
MB_NO_FLAT_1st_GEN	1.300*		−3.097**	−1.736**
	(0.759)		(1.317)	(0.695)
MB_FLAT_2nd_GEN	4.397***	3.097**		1.361
	(1.380)	(1.317)		(1.317)
MB_NO_FLAT_2nd_GEN	3.036***	1.736**	−1.361	
	(0.782)	(0.695)	(1.317)	
Controls	Yes	Yes	Yes	Yes
*N*	2,406	2,406	2,406	2,406
Adjusted *R*^2^	0.075	0.075	0.075	0.075

***p ≤ 0.01; **p ≤ 0.05; *p ≤ 0.10. **Note.** Table 4 reports OLS results (robust standard errors in parentheses). In each model the dependent variable is the metric variable Tax_Progressivity. In each model we jointly apply the classification characteristics of flat tax migration background and generational status to build subgroups of participants MB. In Model 1 MB FLAT 1st GEN participants are the reference group. In Model 2 MB NO FLAT 1st GEN participants are the reference group. In Model 3 MB FLAT 2nd GEN participants are the reference group. In Model 4 MB NO FLAT 2nd GEN participants are the reference group. Controls are not reported here but can be found in Appendix E, Table E2 (Supplementary Material). All variables are defined as described in Appendix C, Table C1 (Supplementary Material).

Overall, the following conclusions are drawn:

**Result 3:**

Among first-generation migrants, participants with flat tax migration backgrounds tend to prefer a lower level of tax progressivity than participants without flat tax migration backgrounds. In contrast, the tax rate progressivity preferences of participants with second-generation flat tax migration backgrounds do not differ from those of second-generation migrants without flat tax migration backgrounds or from those of participants without migration backgrounds.17One potential confounder in the analysis is participants’ real-life income. The analysis of possible income effects reveals that the interaction between income and migration background is significant when comparing individuals with and without a migration background. The findings indicate that as income increases, individuals with a migration background exhibit a stronger preference for tax progression compared to those without a migration background. However, no significant effects are observed within the subsamples of participants with a migration background.

### 4.4 Robustness Check

The previous analysis focused on tax rate progressivity preferences without considering the selected tax rate level.18An overview of the distribution of tax rate levels by subsample can be found in Appendix F, Table F1 (Supplementary Material). However, there is a concern that a higher level of tax rate progressivity is negatively related to the tax rate level of the financially weak group. For instance, a 20 percentage point tax rate progressivity could be more likely when the tax rate for the financially weak group is set at 5 percentage points (range: 5 %–25 %) rather than 20 percentage points (range: 20 %–40 %). Thus, the tax rate level of the financially weak group is a potential explanation for different tax rate progressivity preferences across subgroupings in our sample. To address this concern, we rerun the OLS regression with robust standard errors from our previous analysis. The critical difference is that we now include the tax rate level of the financially weak group as a control variable. The new regression results are shown in Tables E3 and E4 (Appendix E) (Supplementary Material). Table E3 (Supplementary Material), Model 1 provides further evidence for a lower preference for a progressive tax rate among MB participants compared to NO MB participants (Hypothesis 1). In addition, we still detect statistically significant differences between MB 1st GEN participants, MB 2nd GEN participants, and NO MB participants (Hypotheses 3a and 3b) (Table E3 (Supplementary Material), Models 4 and 5). Moreover, we find further support for Hypothesis 2b, which posits that MB NO FLAT participants have a lower preference for a progressive tax rate than NO MB participants (Table E3 (Supplementary Material), Model 3). However, Hypothesis 2a, i.e. that MB FLAT participants have a lower preference for a progressive tax rate than MB NO FLAT participants, cannot withstand the robustness test (Table E3 (Supplementary Material), Model 2). While the previous regression analysis had a statistically significant finding at the 10 % level, the robustness test failed to replicate this statistically significant difference. With respect to further analysis, the results largely remain consistent, but MB FLAT participants are again an exception. While we find a statistically significant difference at the 10 % level between MB FLAT 1st GEN participants and MB NO FLAT 1st GEN participants, the robustness test does not reveal different preferences based on a flat tax migration background (Table E4 (Supplementary Material), Models 1 and 2). In summary, we provide additional support for our primary findings. However, this robustness check does not confirm distinct preferences based on a flat tax migration background.

We also test the hypotheses and conduct further analysis without including control variables (see Tables E5 and E6 in Appendix E (Supplementary Material)). The findings remain essentially unchanged, with one difference in the further analysis; i.e. we could no longer identify a statistically significant distinction between MB_NO_FLAT_1st_GEN and MB_NO_FLAT_2nd_GEN participants (Table E6 (Supplementary Material), Models 2 and 4).

## 5 Conclusions

This paper investigates the relationship between tax culture and personal income tax rate preferences. For this purpose, this study uses the so-called epidemiological approach; i.e. migrants are used to isolate cultural factors. While the literature examining tax culture relies on survey data, we use a novel research design. Our vignette study allows us to overcome the typical drawbacks of laboratory experiments and thus combines the advantages of surveys and experimental research. The participants’ task was to arbitrarily adjust the tax rate of a financially strong group and a financially weak group in an invented region in Germany. We measure tax rate structure preferences based on tax progressivity, which we calculate using the difference between the tax rates of the groups.

One research question of this paper addresses the general impact of tax culture on tax rate structure preferences. Based on policy-feedback theory, we hypothesize that our participants’ tax culture is anchored in the tax scales of their country of origin. Overall, our results provide strong evidence for the general influence of tax culture on our participants’ tax rate structure preferences. This observation aligns with the literature (Alesina and Giuliano 2011; Hedegaard 2023; Luttmer and Singhal 2011). First, our results reveal that participants without a migration background prefer a more progressive income tax rate than participants with a migration background. Thus, the participants’ preferences correspond to their real-life conditions, according to which Germany has a more progressive income tax rate than its ten most significant migration countries. Second, our results suggest that participants with a migration background from a flat tax country prefer lower levels of progressivity than the remaining participants with a migration background. Overall, these results reveal that migrants bring the tax culture from their home countries to their destination countries.

The second research question of this paper addresses whether there is a tax cultural assimilation of individuals with a migration background over time.

Overall, our results indicate that preferences of participants with a migration background converge those of the host society within one generation, In our study, second-generation migrants prefer a more progressive income tax rate than first-generation migrants but a less progressive rate than participants without a migration background. From this result, we conclude that the assimilation process occurs but lasts longer than two generations on average. This result is consistent with the observations of Luttmer and Singhal (2011). However, our results suggest that the duration of the tax cultural assimilation process varies by migrants’ country of origin. Specifically, participants with a flat tax migration background tend to align their progressivity preferences with those of the host society from the first to the second generation. Conversely, while participants with a nonflat tax migration background also adapt their preferences to those of the host society from the first to the second generation, their degree of alignment is less pronounced.

We hope that this paper will encourage future research to gain further insight into tax culture. Based on the findings of this study, future research could address why tax cultural assimilation with the host culture takes a different amount of time depending on migrants’ country of origin. Furthermore, this paper provides insight only into whether the tax rate structure preferences of people with a migration background adjust to non-migrants’ preferences over time. Future studies could examine the reverse situation. It would be interesting to explore whether non-migrants’ preferences adjust to those of persons with a migration background over time. Future research could also consider the perceived social distance (e.g. psychological, social, or political) of migrants to both their country of origin and destination. This would help observe further differences between migrant generations. Moreover, the finding that some of the results do not stand up to the robustness check also reveals a need for further research. One possible explanation would be that individuals migrate due to pre-existing differences in their motivations, such as the desire to earn more money. Migrants may prefer a lower progression of tax rates as they strive for higher incomes. Accordingly, future research could include in the analysis, for example, the reasons for migration, more precise details on household income, and the length of stay in the destination country. Additionally, it would be interesting to examine whether different preferences exist between migrants with one German parent and those with no German parents.

From the results of this study, we can draw three general conclusions. First, tax culture could explain the existence of different tax systems across countries (Luttmer and Singhal 2011). For this reason, researchers should consider the impact of tax culture when contrasting countries in a tax context. Second, migrants are heterogeneous with respect to tax culture. In our study, the tax rate of the country of origin and generational status are found to significantly impact the preferences of participants with a migrant background. Thus, researchers should consider this diversity when selecting samples and interpreting results. The latter aspect is crucial when treating migrants as a uniform group. Furthermore, our work should encourage policymakers to promote the intercultural sensitivity of tax officers and tax authorities. Tax officers should be aware of the potential influence of a specific tax culture and be able to adapt communication to an individual taxpayer’s concerns and preferences accordingly. Third, the diversity of tax culture in modern society will increase due to progressing level of international migration. Consequently, policymakers face the challenge of ensuring the long-term acceptance of the tax system while considering increasingly heterogeneous preferences within the population.

## Supplementary Material

Supplementary Material Details

## Appendix A: Tables on the Structure of Income Tax Systems

See Tables A1 and A2

**Table A1:** Participants’ countries of origin that currently raise or have raised income tax with a flat tax.

Country	Introduction	Abolition	Tax system
Albania	2007	2013	Flat
	2014	Today	Progressive
Armenia	2020	Today	Flat
Belarus	2009	Today	Flat
Bolivia	1986	Today	Flat
Bosnia and Herzegovina	2009	Today	Flat
Bulgaria	2008	Today	Flat
Czech Republic	2008	2012	Flat
	2013	Today	Progressive
Estonia	1994	Today	Flat
Georgia	2005	Today	Flat
Hungary	2011	Today	Flat
Iceland	2007	2009	Flat
	2010	Today	Progressive
Kazakhstan	2007	Today	Flat
Kyrgyzstan	2006	Today	Flat
Latvia	1997	2017	Flat
	2018	Today	Progressive
Lithuania	1994	2018	Flat
	2019	Today	Progressive
Moldova	2018	Today	Flat
Montenegro	2007	2012	Flat
	2013	2019	Progressive
	2019	2021	Flat
	2022	Today	Progressive
Romania	2005	Today	Flat
Russian	2001	2020	Flat
	2021	Today	Progressive
Serbia	2003	2009	Flat
	2010	Today	Progressive
Slovakia	2004	2012	Flat
	2013	Today	Progressive
Turkmenistan	2005	Today	Flat
Ukraine	2004	2010	Flat
	2012	2014	Progressive
	2015	Today	Flat
Uzbekistan	2019	Today	Flat

**Note.** Table A1 shows all countries of origin of our subjects that have levied income tax with a flat tax for at least one year in the past 30 years. The classification of countries did not consider possible special regulations or exceptions to tax law. Due to inconsistent information, Iraq and Tajikistan are not included in the analysis when tax rate preferences are examined in the context of a flat tax. Sources: Balatsky and Ekimova (2021), Bartha (2014), Buvsara and Shokhida (2019), Efremidze and Salayeva (2021), EY (2022), Ilollari and Kripa (2016), IMF (2023), IOTA (2018), ITUC (2022), OECD (2023b), Peichl (2014), PWC (2023), Regional Cooperation Council (2021), Wheaton (2022).

**Table A2:** German income tax rate compared to that of the most important migration countries.

Country	Tax system	Personal income tax
Germany	Progressive	0 %–45 %
Türkiye	Progressive	15 %–40 %
Poland	Progressive	12 %–32 %
Russia	Flat	13 %
Kazakhstan	Flat	10 %
Syria	Progressive	0 %–22 %
Romania	Flat	10 %
Italy	Progressive	23 %–43 %
Bosnia and Herzegovina	Flat	10 %
Kosovo	Progressive	0 %–10 %
Greece	Progressive	9 %–44 %

**Note.** Table A2 shows the ten most represented migration countries in Germany by number of persons (BMI/BAMF 2023, p. 149). The values in italics indicate the range of marginal tax rates for the countries listed. These individual tax rates do not encompass the diverse assortment of unique rates, programs, and exceptions applicable to specific income categories. Information about personal income tax rates can be found in Deloitte (2019), Balatsky and Ekimova (2021), PWC (2023).
