# Supplementary material for: The Impact of Tax Culture on Tax Rate Structure Preferences: Results from a Vignette Study with Migrants and Non-Migrants in Germany
Source: Rev Law Econ. 2025 Jun 6;21(2):443–78. doi: 10.1515/rle-2024-0041 (PMC12452228; doi:10.1515/rle-2024-0041)
Supplement: Supplementary file 1 — Supplementary Material Details [file j_rle-2024-0041_suppl_001.docx]

**Supplementary Material for “The Impact of Tax Culture on Tax Rate Structure Preferences: Results from a Vignette Study with Migrants and Non-migrants in Germany”**

**by Dirk Kiesewetter and André Machwart**

**Appendix B Components of the Vignette Study**

1. ***Vignette Example***

Hint: This vignette example shows a possible scenario for a participant without a migration background. This vignette study examined the link between taxation and migration in multifaceted ways. Therefore, not all information in this vignette is relevant to this study.

All vignettes start identically:

The following is a description of a region in Germany that we have entirely created. While the mentioned statistics are fabricated, they may resemble those of actual regions in Germany. Please carefully review the description, as we will be asking you questions about it later.

Please imagine that you live in a region in Germany where a total of 60 thousand people live. Of these 60 thousand people, 30 thousand have no migration background and 30 thousand have a migration background.
A person has a migration background if a person himself or at least one of its parents was not born in Germany.

Assignment of the individual migration status within this fictitious region (mirrored the participant’s migration status in real life):

- From your personal information at the beginning of this study, it is clear that in reality you do not have a migration background. Therefore, please assume that you do not have a migration background in this region in Germany.


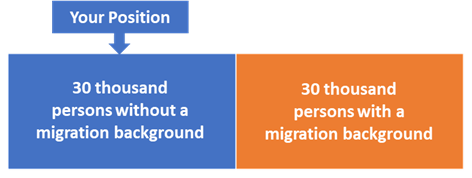


Division of the fictitious population into a financially weak group and a financially strong group:

In a brochure you can see a picture that divides the people of this region in Germany into the financially weak and the financially strong. Here, 1 icon character represents exactly 1 thousand persons. Important is:

- There are 30 thousand financially weak and 30 thousand financially strong (30 icons each).

The following bullet points in italics are not relevant for the purpose of this study:

- *Out of 30 financially weak persons, 5 persons have no migration background, and 25 persons have a migration background.*
- *Out of 30 financially strong persons, 25 persons have no migration background, and 5 persons have a migration background.*

Assignment of the participant’s individual financial status within this fictitious region:

- Please assume that you are a financially strong person in this region in Germany with your monthly disposable income.


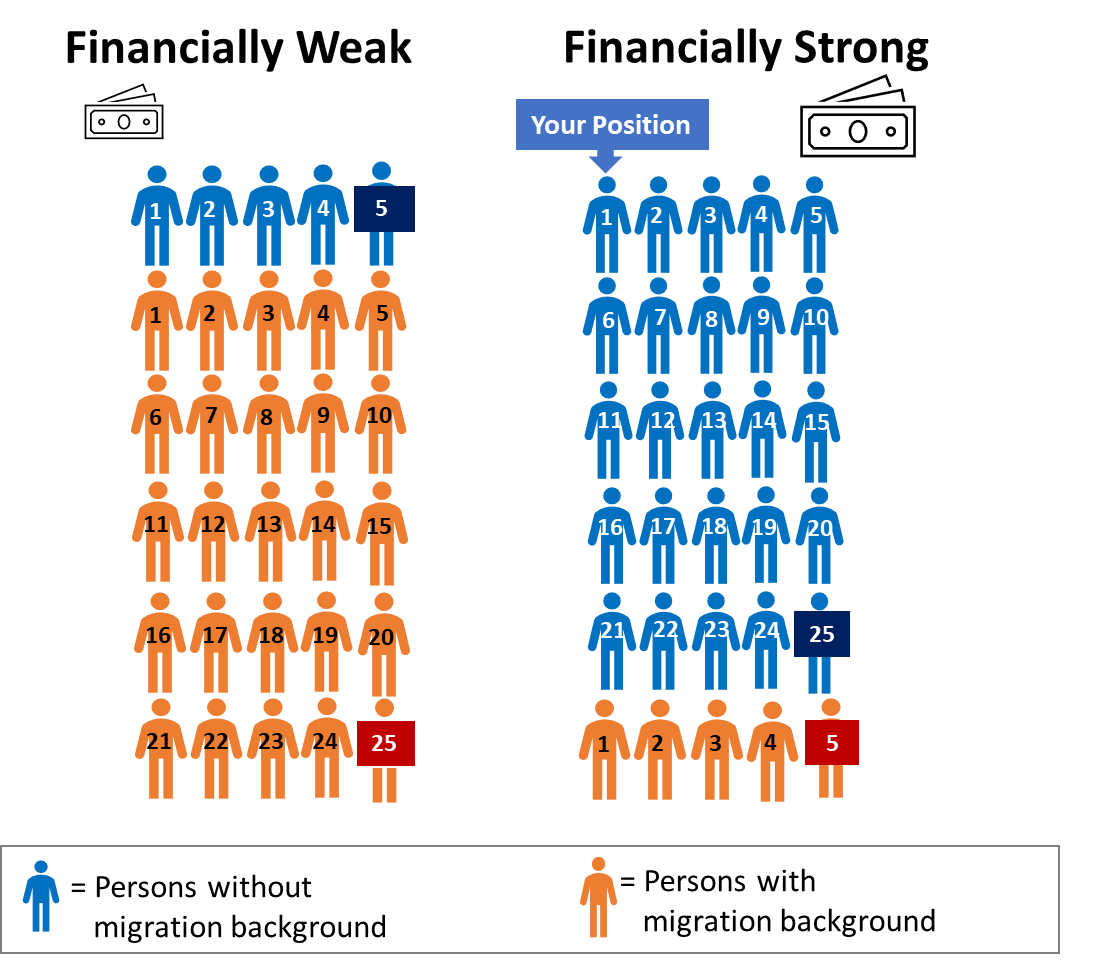


1. ***Comprehension Questions***
2. Do you have a migration background, or do you not have a migration background in this region in Germany?

☐ I have a migration background

☐ I do not have a migration background

1. According to the description of this region in Germany, are you a financially weak person or are you a financially strong person?

☐ I am a financially weak person

☐ I am a financially strong person

1. According to the description of this region in Germany, do the other people with a migration background have exactly the same migration background as you or different migration backgrounds?

☐ The other persons with a migration background have exactly the same migration background as me

☐ The other persons with a migration background have different migration backgrounds

1. According to the description of this region in Germany, are more people with a migration background or more people without a migration background financially weak?

☐ More persons without a migration background are financially weak than persons with a migration background

☐ More persons with a migration background are financially weak than persons without a migration background

1. ***The Task of the Participants***

Another much-discussed topic in this region in Germany is the collection of tax revenues. We are interested in how you think tax revenues should be collected in this region. For this reason, you are now asked what percentage of gross annual income the financially strong, the financially weak, and you personally should pay in income tax in this region in Germany.

Hint:

All tax rates refer to the average income tax rate. The average income tax rate indicates how many cents, on average, have to be paid to the tax office out of every euro earned.

The financially strong currently have to pay 25% of the gross annual income in this region in Germany in income tax.
What is your opinion on the income tax rate? What percentage do you think is appropriate for the financially strong in this region?

| ________ | % |
| --- | --- |

The financially weak currently have to pay 10% of the gross annual income in this region in Germany in income tax.
What is your opinion on the income tax rate? What percentage do you think is appropriate for the financially strong in this region?

| ________ | % |
| --- | --- |

You personally currently have to pay (10% / 25%) of the gross annual income in this region in Germany in income tax.^^[[1]](#footnote-1)^^
What is your opinion on the income tax rate? What percentage do you think is appropriate for you personally in this region?

| ________ | % |
| --- | --- |

1. ***Questionnaires***

Ex ante questionnaire

(1) What is your age (in years)?

[Dropdown list from 6 to 100 years]

(2) Are you female, male, or diverse?

| ☐ Female | ☐ Male | ☐ Diverse |
| --- | --- | --- |

(3) What is your marital status?

| ☐ Single | ☐ Married / Registered civil partnership |
| --- | --- |
| ☐ Divorced / Widowed | ☐ Other |

(4) In which country were you born?

[Auto-complete field with list of all countries]

(5) In which country was your mother born?

[Auto-complete field with list of all countries]

(6) In which country was your father born?

[Auto-complete field with list of all countries]

(7) If you add up what remains after deduction of taxes and social security from your own income and, if applicable, from the income of your spouse or partner (net household income), what is this amount?

| ☐ under 900 € | ☐ 900 € to under 1,300 € |
| --- | --- |
| ☐ 1,300 € to under 1,500 € | ☐ 1,500 € to under 2,000 € |
| ☐ 2,000 € to under 2,600 € | ☐ 2,600 € to under 3,600 € |
| ☐ 3,600 € to under 5,000 € | ☐ more than 5,000 € |

(8) Please indicate the highest level of education you have achieved.
*If you obtained your highest educational qualification abroad, please indicate to which educational qualification in Germany your educational qualification is comparable.*

| ☐ Hauptschulabschluss |  | ☐ Realschulabschluss |
| --- | --- | --- |
| ☐ Hochschulreife |  | ☐ Bachelor or equivalent |
| ☐ Master or equivalent |  | ☐ Doctoral degree |
| ☐ Other |  |  |

Ex post questionnaire

(9) What is your citizenship?

[Auto-complete field with list of all countries]

(10) Do you have another citizenship? If yes, which one?
*If you do not have another citizenship, please enter “No”.*

[Auto-complete field with list of all countries]

(11) Are you employed now or not?
*Please select all the items in this list that apply to you. You can also select more than one answer.*

| ☐ 30 hours a week or more |
| --- |
| ☐ Less than 30 hours a week |
| ☐ Self-employed |
| ☐ Civil servant |
| ☐ Retired, pensioned |
| ☐ Housewife, houseman without other employment |
| ☐ Pupil, student, in vocational training |
| ☐ Unemployed |
| ☐ Unable to work due to a disability |

(12) How willing are you to take risks, in general?

| Not willing to take risks at all | | | |  |  |  | Very willing to take risks | | |
| --- | --- | --- | --- | --- | --- | --- | --- | --- | --- |
| 1 | 2 | 3 | 4 | 5 | 6 | 7 | 8 | 9 | 10 |
| ☐ | ☐ | ☐ | ☐ | ☐ | ☐ | ☐ | ☐ | ☐ | ☐ |

Please indicate for each of the following actions whether you think it can always be justified, never be justified, or something in between.

|  |  | Never  justified | | | |  |  | Always  justified | | | |
| --- | --- | --- | --- | --- | --- | --- | --- | --- | --- | --- | --- |
|  |  | 1 | 2 | 3 | 4 | 5 | 6 | 7 | 8 | 9 | 10 |
| (13) | Avoiding a fare on public transport | ☐ | ☐ | ☐ | ☐ | ☐ | ☐ | ☐ | ☐ | ☐ | ☐ |
| (14) | Claiming government benefits to which you are not entitled | ☐ | ☐ | ☐ | ☐ | ☐ | ☐ | ☐ | ☐ | ☐ | ☐ |
| (15) | False declaration of assets or income in order to receive state benefits (e.g., student loans, unemployment benefits) | ☐ | ☐ | ☐ | ☐ | ☐ | ☐ | ☐ | ☐ | ☐ | ☐ |

On this list you can see opposing opinions on different topics. Where would you place your own views on this scale?
*The further to the left (smartphone: top) you select, the more the left (smartphone: top) view applies to you, the further to the right (smartphone: bottom) you select, the more the right (smartphone: bottom) view applies to you.*

|  |  | 5 | 4 | 3 | 2 | 1 | 1 | 2 | 3 | 4 | 5 |  |
| --- | --- | --- | --- | --- | --- | --- | --- | --- | --- | --- | --- | --- |
| (16) | Incomes should be made more equal | ☐ | ☐ | ☐ | ☐ | ☐ | ☐ | ☐ | ☐ | ☐ | ☐ | There should be greater incentives for individual effort |
| (17) | Hard work doesn’t generally bring success – it’s more a matter of luck and connections | ☐ | ☐ | ☐ | ☐ | ☐ | ☐ | ☐ | ☐ | ☐ | ☐ | In the long run, hard work usually brings a better life |
| (18) | The German government should take more responsibility to ensure that everyone is provided for | ☐ | ☐ | ☐ | ☐ | ☐ | ☐ | ☐ | ☐ | ☐ | ☐ | People should take more responsibility to provide for themselves |
|  |  |  |  |  |  |  |  |  |  |  |  |  |

(19) How would you rate your knowledge of the German tax system?

| None at all |  |  | Above average | |
| --- | --- | --- | --- | --- |
| 1 | 2 | 3 | 4 | 5 |
| ☐ | ☐ | ☐ | ☐ | ☐ |

For each of the following points, please indicate whether you fully agree, do not agree at all, or something in between.

|  |  | Do not agree  at all | | | |  |  | Fully agree | | | |
| --- | --- | --- | --- | --- | --- | --- | --- | --- | --- | --- | --- |
|  |  | 1 | 2 | 3 | 4 | 5 | 6 | 7 | 8 | 9 | 10 |
| (20) | I have great trust in the state and its state institutions. | ☐ | ☐ | ☐ | ☐ | ☐ | ☐ | ☐ | ☐ | ☐ | ☐ |
| (21) | The German state and its state institutions have extensive means at their disposal to combat tax evasion and enforce tax honesty. | ☐ | ☐ | ☐ | ☐ | ☐ | ☐ | ☐ | ☐ | ☐ | ☐ |
| (22) | When I talk about people without migration background I usually say “we” rather than “they”. | ☐ | ☐ | ☐ | ☐ | ☐ | ☐ | ☐ | ☐ | ☐ | ☐ |
| (23) | When I talk about people with migration background I usually say “we” rather than “they”. | ☐ | ☐ | ☐ | ☐ | ☐ | ☐ | ☐ | ☐ | ☐ | ☐ |
| (24) | When I talk about people who have exactly the identical migration background as me, I usually say “we” and not “they”. | ☐ | ☐ | ☐ | ☐ | ☐ | ☐ | ☐ | ☐ | ☐ | ☐ |
| (25) | When someone says something bad about people without a migration background, I feel like they said something bad about me. | ☐ | ☐ | ☐ | ☐ | ☐ | ☐ | ☐ | ☐ | ☐ | ☐ |
| (26) | When someone says something bad about people with a migration background, I feel like they said something bad about me. | ☐ | ☐ | ☐ | ☐ | ☐ | ☐ | ☐ | ☐ | ☐ | ☐ |
| (27) | When someone says something bad about people who have exactly the identical migration background as me, I feel like they said something bad about me. | ☐ | ☐ | ☐ | ☐ | ☐ | ☐ | ☐ | ☐ | ☐ | ☐ |
| (28) | Only people who have lived in Germany for a minimum number of years should receive state benefits. | ☐ | ☐ | ☐ | ☐ | ☐ | ☐ | ☐ | ☐ | ☐ | ☐ |

(29) Just in your opinion, how do the economic opportunities of people with a migration background compare to the economic opportunities of people without a migration background. Do people with a migration background get many fewer opportunities, about the same number, or many more opportunities than people without a migration background.

| Many fewer economic  opportunities | | | About the same number of  economic opportunities | | |  | Many more  economic opportunities | |
| --- | --- | --- | --- | --- | --- | --- | --- | --- |
| 4 | 3 | 2 | 1 | 0 | 1 | 2 | 3 | 4 |
| ☐ | ☐ | ☐ | ☐ | ☐ | ☐ | ☐ | ☐ | ☐ |

Please indicate how close you do feel to the following persons.

|  |  | Very close | Close | Not very close | Not close at all |
| --- | --- | --- | --- | --- | --- |
| (30) | How close do you feel to people without a migration background in Germany? | ☐ | ☐ | ☐ | ☐ |
| (31) | How close do you feel to people with a migration background in Germany? | ☐ | ☐ | ☐ | ☐ |
| (32) | How close do you feel to people who have exactly the identical migration background as you in Germany? | ☐ | ☐ | ☐ | ☐ |

(33) How proud are you to live in Germany?

| Very proud | Quite proud | Not very proud | Not at all proud |
| --- | --- | --- | --- |
| ☐ | ☐ | ☐ | ☐ |

(34) Apart from unavoidable contacts (e.g., at work, at the educational institution, in your own household, etc.): How often do you socialize with people who were not born in Germany? This refers to personal meetings, not just greetings.

| ☐ Every day | ☐ Several times a week |
| --- | --- |
| ☐ Once a week | ☐ Several times a month |
| ☐ Rarely | ☐ Not at all |

(35) Apart from unavoidable contacts (e.g., at work, at the educational institution, in your own household, etc.): How often do you socialize with people who were born in Germany? This refers to personal meetings, not just greetings.

| ☐ Every day | ☐ Several times a week |
| --- | --- |
| ☐ Once a week | ☐ Several times a month |
| ☐ Rarely | ☐ Not at all |

(36) What language do you most often speak at home?

| ☐ Arabic | ☐ German | ☐ English | ☐ French |
| --- | --- | --- | --- |
| ☐ Italian | ☐ Polish | ☐ Kazakh | ☐ Kurdish |
| ☐ Romanian | ☐ Russian | ☐ Spanish | ☐ Turkish |
| ☐ Other European language | | ☐ Other African language | |
| ☐ Other Asian language | | ☐ Other language | |

(37) How secure do you think your current job is?

| ☐ Very secure | ☐ Relatively secure |
| --- | --- |
| ☐ Not very secure | ☐ Not secure at all |
| ☐ I am not employed |  |

(38) In political matters, people talk of “the left” and “the right.” How would you place your views on this scale, generally speaking?
*The further to the left (smartphone: top) you select, the more “Left” applies to you, the further to the right (smartphone: bottom) you select, the more “Right” applies to you.*

| Left | |  |  |  |  |  |  | Right | |
| --- | --- | --- | --- | --- | --- | --- | --- | --- | --- |
| 5 | 4 | 3 | 2 | 1 | 1 | 2 | 3 | 4 | 5 |
| ☐ | ☐ | ☐ | ☐ | ☐ | ☐ | ☐ | ☐ | ☐ | ☐ |

(39) How often do you pray besides weddings, funerals, and baptisms?

| ☐ Several times a day | ☐ Once a day |
| --- | --- |
| ☐ Several times each week | ☐ Several times a month |
| ☐ Only when attending religious services | ☐ Once a year |
| ☐ Less often | ☐ Never, practically never |

(40) How satisfied are you with the financial situation of your household?

| Not satisfied at all | | | |  |  |  | Fully satisfied | | |
| --- | --- | --- | --- | --- | --- | --- | --- | --- | --- |
| 1 | 2 | 3 | 4 | 5 | 6 | 7 | 8 | 9 | 10 |
| ☐ | ☐ | ☐ | ☐ | ☐ | ☐ | ☐ | ☐ | ☐ | ☐ |

(41) Do you feel that you belong to a particular ethnic group? If yes, which one?
*Please enter the ethnic group to which you feel you belong. If you do not feel that you belong to any ethnic group, please enter “No”.*

[Text field]

(42) Please think about the region in which you currently live. How many out of 100 people do you think have a migration background?
*The number of persons must assume a value between 0 and 100.*

[Text field]

(43) If you think about the invented region and your decisions in the context of this study, did you think of a particular region in Germany during the conduction? If yes, which one?
*If you had a specific region in Germany in mind, please enter it in the field. If you did not have a specific region in mind, please enter “No”.*

[Text field]

(44) If you had a specific region in Germany in mind while conducting this study, is your attitude towards this region very positive, positive, neutral, negative, or very negative?

| ☐ Very positive | ☐ Positive |
| --- | --- |
| ☐ Neutral | ☐ Negative |
| ☐ Very negative | ☐ I have not thought of any particular region |

**Appendix C Definitions of the Variables**

**Table C1.** Overview of all variables.

| **Dependent variable** | |
| --- | --- |
| Tax_Progressivity | Metric variable that captures the difference between the tax rate of the financially strong group and the financially weak group of the (fictitious) society in the decision-making situation. |
| **Independent variables** | |
| MB | Dummy equal to 1 if the respondent or at least one of his or her parents was not born in Germany, otherwise 0. |
| MB_1st_GEN | Dummy equal to 1 if the respondent was not born in Germany, otherwise 0. |

(Continued)

| MB_2nd_GEN | Dummy equal to 1 if the respondent was born in Germany and at least one of his or her parents was not born in Germany, otherwise 0. | |
| --- | --- | --- |
| MB_FLAT | Dummy equal to 1 if the respondent or at least one of his or her parents was born in a country that levied income tax with a flat tax for at least one year in the past 30 years, otherwise 0. | |
| MB_FLAT_1st_GEN | Dummy equal to 1 if the respondent was born in a country that levied income tax with a flat tax for at least one year in the past 30 years, otherwise 0. | |
| MB_FLAT_2nd_GEN | Dummy equal to 1 if the respondent was born in Germany and at least one of his or her parents was born in a country that levied income tax with a flat tax for at least one year in the past 30 years, otherwise 0. | |
| MB_NO_FLAT | Dummy equal to 1 if the respondent has a migration background and the respondent and his or her parents were not born in a country that levied income tax with a flat tax for at least one year in the past 30 years, otherwise 0. | |
| MB_NO_FLAT_1st_GEN | Dummy equal to 1 if the respondent was not born in Germany but not in a country that levied income tax with a flat tax for at least one year in the past 30 years, otherwise 0. | |
| MB_NO_FLAT_2nd_GEN | Dummy equal to 1 if the respondent was born in Germany and at least one of his or her parents was not born in Germany but not in a country that levied income tax with a flat tax for at least one year in the past 30 years, otherwise 0. | |
| NO_MB | Dummy equal to 1 if the respondent and his or her parents were born in Germany, otherwise 0. | |
| **Control variables** | | |
| Academic | | Dummy equal to 1 if the participant has achieved an academic qualification, 0 otherwise. |
| Age | | Variable collects the age (in years) of a participant. |
| Female | | Dummy equal to 1 if the participant is female, 0 otherwise. |
| Financial_Strain | | Perceived financial satisfaction, measured on a 10-point scale (1 = “Not satisfied at all” to 10 = “Fully satisfied”). |
| High_Income | | Differentiation between eight (1 to 8) categories, which sort the income in ascend-ing order of amount: (1) under 900 €; (2) 900 € to under 1,300 €; (3) 1,300 € to under 1,500 €; (4) 1,500 € to under 2,000 €; (5) 2,000 € to under 2,600 €; (6) 2,600 € to under 3,600 €; (7) 3,600 € to under 5,000 €; (8) more than 5,000 €. Dummy equal to 1 if the participant states to earn according to category (7) or (8), otherwise 0. |
| Job_is_Secure | | Dummy equal to 1 if the participant perceives his current job as very secure or relatively secure, otherwise 0. |

(Continued)

| Middle_Income | Differentiation between eight (1 to 8) categories, which sort the income in ascend-ing order of amount: (1) under 900 €; (2) 900 € to under 1,300 €; (3) 1,300 € to under 1,500 €; (4) 1,500 € to under 2,000 €; (5) 2,000 € to under 2,600 €; (6) 2,600 € to under 3,600 €; (7) 3,600 € to under 5,000 €; (8) more than 5,000 €. Dummy equal to 1 if the participant states to earn according to category (5) or (6), otherwise 0. |
| --- | --- |
| No_Faith_in_Social_ Mobility | Perceived chance of social mobility, measured on a 10-point scale (1= “In the long run, hard work usually brings a better life” to 10 = “Hard work doesn’t generally bring success – it’s more a matter of luck and connections”). |
| Occupation_Status | Dummy equal to 1 if the participant is employed full-time, part-time, or self-employed, otherwise 0. |
| Pro_Income_Equalization | Attitude toward income equalization, measured on a 10-point scale (1= “There should be greater incentives for individual effort” to 10 = “Incomes should be made more equal”). |
| Religious | Dummy equal to 1 if the participant prays at least during participation in religious services, otherwise 0. |
| Right_Orientation | Perceived political attitude, measured by a dummy equal to 1 if a respondent rates his or her political attitude on a 10-point scale (1 = “Left” to 10 = “Right”) as at least with the value 6, otherwise 0. |
| Risk_Attitude | Perception of the own risk attitude, measured on a 10-point scale (1 = “Not willing to take risks at all” to 10 = “Very willing to take risks”). |
| Tax_Knowledge | Perceived knowledge of the German tax system, measured on a 5-point scale (1= “None at all” to 5= “Above average”). |
| Trust_in_State | Participants’ personal evaluation of the statement “I have great trust in the state and its state institutions.”, measured by 10-point scale (1 = “Do not agree at all” to 10 = “Fully agree”). |
| **Other variables** | |
| Income | Differentiation between eight (1 to 8) categories, which sort the income in ascend-ing order of amount:  (1) under 900 €; (2) 900 € to under 1,300 €; (3) 1,300 € to under 1,500 €; (4) 1,500 € to under 2,000 €; (5) 2,000 € to under 2,600 €; (6) 2,600 € to under 3,600 €; (7) 3,600 € to under 5,000 €; (8) more than 5,000 € |
| Tax_Progressivity_Ordinal | Ordinal variable that captures the difference between the tax rate of the financially strong group and the financially weak group of the (fictitious) society in the decision-making situation: (0) Tax Progressivity < 15%; (1) Tax Progressivity = 15%; (2) Tax Progressivity > 15%. |
| Tax_Rate_Strong_Group | Metric variable that captures the tax rate of the financially strong group of the (fictitious) society in the decision-making situation. |
| Tax_Rate_Weak_Group | Metric variable that captures the tax rate of the financially weak group of the (fictitious) society in the decision-making situation. |

**Appendix D Summary statistics**

**Table D1.** Summary statistics of control variables – NO MB participants, MB participants, and total sample.

|  | NO_MB | | | | | MB | | | | | Total | | | | |
| --- | --- | --- | --- | --- | --- | --- | --- | --- | --- | --- | --- | --- | --- | --- | --- |
|  | Mean | SD | Min. | Max. | N | Mean | SD | Min. | Max. | N | Mean | SD | Min. | Max. | N |
| Age | 41.51 | 14.23 | 18 | 84 | 840 | 34.10 | 11.21 | 18 | 72 | 1,582 | 36.67 | 12.83 | 18 | 84 | 2,422 |
| Female | 0.48 | 0.50 | 0 | 1 | 840 | 0.61 | 0.49 | 0 | 1 | 1,582 | 0.57 | 0.50 | 0 | 1 | 2,422 |
| Academic | 0.33 | 0.47 | 0 | 1 | 840 | 0.36 | 0.48 | 0 | 1 | 1,582 | 0.35 | 0.48 | 0 | 1 | 2,422 |
| Occupation_Status | 0.75 | 0.43 | 0 | 1 | 840 | 0.73 | 0.44 | 0 | 1 | 1,582 | 0.74 | 0.44 | 0 | 1 | 2,422 |
| Middle_Income | 0.33 | 0.47 | 0 | 1 | 840 | 0.33 | 0.47 | 0 | 1 | 1,582 | 0.33 | 0.47 | 0 | 1 | 2,422 |
| High_Income | 0.29 | 0.46 | 0 | 1 | 840 | 0.25 | 0.43 | 0 | 1 | 1,582 | 0.26 | 0.44 | 0 | 1 | 2,422 |
| Financial_Strain | 5.29 | 2.55 | 1 | 10 | 840 | 5.18 | 2.52 | 1 | 10 | 1,582 | 5.22 | 2.53 | 1 | 10 | 2,422 |
| Risk_Attitude | 5.09 | 2.19 | 1 | 10 | 840 | 5.34 | 2.35 | 1 | 10 | 1,582 | 5.25 | 2.30 | 1 | 10 | 2,422 |
| Tax_Knowledge | 2.83 | 0.96 | 1 | 5 | 840 | 2.90 | 0.98 | 1 | 5 | 1,582 | 2.88 | 0.98 | 1 | 5 | 2,422 |
| Pro_Income_Equalization | 5.48 | 2.92 | 1 | 10 | 840 | 5.58 | 2.92 | 1 | 10 | 1,578 | 5.55 | 2.92 | 1 | 10 | 2,418 |
| No_Faith_in_Social_Mobility | 5.81 | 2.83 | 1 | 10 | 840 | 6.07 | 2.88 | 1 | 10 | 1,580 | 5.98 | 2.86 | 1 | 10 | 2,420 |
| Trust_in_State | 4.35 | 2.52 | 1 | 10 | 839 | 4.61 | 2.58 | 1 | 10 | 1,578 | 4.52 | 2.56 | 1 | 10 | 2,417 |
| Job_is_Secure | 0.73 | 0.44 | 0 | 1 | 840 | 0.71 | 0.45 | 0 | 1 | 1,582 | 0.72 | 0.45 | 0 | 1 | 2,422 |
| Right_Orientation | 0.41 | 0.49 | 0 | 1 | 840 | 0.30 | 0.46 | 0 | 1 | 1,582 | 0.34 | 0.47 | 0 | 1 | 2,422 |
| Religious | 0.35 | 0.48 | 0 | 1 | 840 | 0.52 | 0.50 | 0 | 1 | 1,582 | 0.46 | 0.50 | 0 | 1 | 2,422 |

**Note.** Table D1 provides a summary statistic of the control variables of our OLS analysis. A distinction is made between the NO MB participants and the MB participants. In addition, the total sample is considered. This table provides information regarding the mean value (Mean), the standard deviation (SD), the minimum expression (Min.), and the maximum expression (Max.) of a variable. Moreover, the number of observations (N) is reported. All variables are defined as described in Appendix C, Table C1.

**Table D2.** Summary statistics of control variables – MB FLAT, MB NO FLAT, MB 1st GEN, and MB 2nd GEN participants.

|  | MB_FLAT | | | | | MB_NO_FLAT | | | | | MB_1st_GEN | | | | | MB_2nd_GEN | | | | |
| --- | --- | --- | --- | --- | --- | --- | --- | --- | --- | --- | --- | --- | --- | --- | --- | --- | --- | --- | --- | --- |
|  | Mean | SD | Min. | Max. | N | Mean | SD | Min. | Max. | N | Mean | SD | Min. | Max. | N | Mean | SD | Min. | Max. | N |
| Age | 33.83 | 10.69 | 18 | 69 | 424 | 34.26 | 11.40 | 18 | 72 | 1,152 | 36.46 | 11.29 | 18 | 72 | 900 | 30.98 | 10.32 | 18 | 69 | 682 |
| Female | 0.70 | 0.46 | 0 | 1 | 424 | 0.58 | 0.49 | 0 | 1 | 1,152 | 0.59 | 0.49 | 0 | 1 | 900 | 0.64 | 0.48 | 0 | 1 | 682 |
| Academic | 0.38 | 0.49 | 0 | 1 | 424 | 0.35 | 0.48 | 0 | 1 | 1,152 | 0.40 | 0.49 | 0 | 1 | 900 | 0.30 | 0.46 | 0 | 1 | 682 |
| Occupation_Status | 0.72 | 0.45 | 0 | 1 | 424 | 0.73 | 0.44 | 0 | 1 | 1,152 | 0.74 | 0.44 | 0 | 1 | 900 | 0.72 | 0.45 | 0 | 1 | 682 |
| Middle_Income | 0.34 | 0.47 | 0 | 1 | 424 | 0.33 | 0.47 | 0 | 1 | 1,152 | 0.32 | 0.47 | 0 | 1 | 900 | 0.34 | 0.48 | 0 | 1 | 682 |
| High_Income | 0.25 | 0.43 | 0 | 1 | 424 | 0.25 | 0.43 | 0 | 1 | 1,152 | 0.24 | 0.43 | 0 | 1 | 900 | 0.26 | 0.44 | 0 | 1 | 682 |
| Financial_Strain | 5.16 | 2.39 | 1 | 10 | 424 | 5.20 | 2.57 | 1 | 10 | 1,152 | 5.23 | 2.52 | 1 | 10 | 900 | 5.12 | 2.51 | 1 | 10 | 682 |
| Risk_Attitude | 5.29 | 2.41 | 1 | 10 | 424 | 5.36 | 2.32 | 1 | 10 | 1,152 | 5.23 | 2.49 | 1 | 10 | 900 | 5.49 | 2.15 | 1 | 10 | 682 |
| Tax_Knowledge | 2.88 | 0.99 | 1 | 5 | 424 | 2.91 | 0.99 | 1 | 5 | 1,152 | 2.89 | 1.00 | 1 | 5 | 900 | 2.92 | 0.96 | 1 | 5 | 682 |
| Pro_Income_Equalization | 5.31 | 3.00 | 1 | 10 | 423 | 5.67 | 2.89 | 1 | 10 | 1,149 | 5.53 | 2.94 | 1 | 10 | 896 | 5.65 | 2.89 | 1 | 10 | 682 |
| No_Faith_in_Social_Mobility | 6.12 | 2.96 | 1 | 10 | 424 | 6.05 | 2.85 | 1 | 10 | 1,151 | 5.92 | 2.91 | 1 | 10 | 899 | 6.26 | 2.83 | 1 | 10 | 681 |
| Trust_in_State | 4.71 | 2.65 | 1 | 10 | 423 | 4.56 | 2.55 | 1 | 10 | 1,149 | 5.08 | 2.62 | 1 | 10 | 897 | 3.98 | 2.38 | 1 | 10 | 681 |
| Job_is_Secure | 0.71 | 0.46 | 0 | 1 | 424 | 0.71 | 0.45 | 0 | 1 | 1,152 | 0.69 | 0.46 | 0 | 1 | 900 | 0.74 | 0.44 | 0 | 1 | 682 |
| Right_Orientation | 0.36 | 0.48 | 0 | 1 | 424 | 0.28 | 0.45 | 0 | 1 | 1,152 | 0.35 | 0.48 | 0 | 1 | 900 | 0.24 | 0.43 | 0 | 1 | 682 |
| Religious | 0.51 | 0.50 | 0 | 1 | 424 | 0.53 | 0.50 | 0 | 1 | 1,152 | 0.53 | 0.50 | 0 | 1 | 900 | 0.52 | 0.50 | 0 | 1 | 682 |

**Note.** Table D2 provides a summary statistic of the control variables of our OLS analysis. A distinction is made between MB FLAT participants, MB NO FLAT participants, MB 1st GEN participants, and MB 2nd GEN participants. This table provides information regarding the mean value (Mean), the standard deviation (SD), the minimum expression (Min.), and the maximum expression (Max.) of a variable. Moreover, the number of observations (N) is reported. All variables are defined as described in Appendix C, Table C1.

**Table D3.** Summary statistics of control variables – further analysis.

|  | MB_FLAT_1st_GEN | | | | | MB_NO_FLAT_1st_GEN | | | | | MB_FLAT_2nd_GEN | | | | | MB_NO_FLAT_2nd_GEN | | | | |
| --- | --- | --- | --- | --- | --- | --- | --- | --- | --- | --- | --- | --- | --- | --- | --- | --- | --- | --- | --- | --- |
|  | Mean | SD | Min. | Max. | N | Mean | SD | Min. | Max. | N | Mean | SD | Min. | Max. | N | Mean | SD | Min. | Max. | N |
| Age | 34.73 | 10.05 | 18 | 68 | 324 | 37.53 | 11.81 | 18 | 72 | 572 | 30.92 | 12.15 | 18 | 69 | 100 | 31.03 | 9.98 | 18 | 67 | 580 |
| Female | 0.71 | 0.45 | 0 | 1 | 324 | 0.52 | 0.50 | 0 | 1 | 572 | 0.66 | 0.48 | 0 | 1 | 100 | 0.64 | 0.48 | 0 | 1 | 580 |
| Academic | 0.40 | 0.49 | 0 | 1 | 324 | 0.41 | 0.49 | 0 | 1 | 572 | 0.32 | 0.47 | 0 | 1 | 100 | 0.30 | 0.46 | 0 | 1 | 580 |
| Occupation_Status | 0.75 | 0.44 | 0 | 1 | 324 | 0.74 | 0.44 | 0 | 1 | 572 | 0.65 | 0.48 | 0 | 1 | 100 | 0.73 | 0.45 | 0 | 1 | 580 |
| Middle_Income | 0.35 | 0.48 | 0 | 1 | 324 | 0.30 | 0.46 | 0 | 1 | 572 | 0.29 | 0.46 | 0 | 1 | 100 | 0.35 | 0.48 | 0 | 1 | 580 |
| High_Income | 0.24 | 0.43 | 0 | 1 | 324 | 0.24 | 0.43 | 0 | 1 | 572 | 0.27 | 0.45 | 0 | 1 | 100 | 0.25 | 0.44 | 0 | 1 | 580 |
| Financial_Strain | 5.23 | 2.43 | 1 | 10 | 324 | 5.25 | 2.58 | 1 | 10 | 572 | 4.92 | 2.27 | 1 | 9 | 100 | 5.15 | 2.56 | 1 | 10 | 580 |
| Risk_Attitude | 5.23 | 2.53 | 1 | 10 | 324 | 5.23 | 2.46 | 1 | 10 | 572 | 5.50 | 1.96 | 1 | 10 | 100 | 5.48 | 2.17 | 1 | 10 | 580 |
| Tax_Knowledge | 2.91 | 1.00 | 1 | 5 | 324 | 2.89 | 1.01 | 1 | 5 | 572 | 2.77 | 0.93 | 1 | 5 | 100 | 2.94 | 0.97 | 1 | 5 | 580 |
| Pro_Income_Equalization | 5.26 | 3.02 | 1 | 10 | 323 | 5.67 | 2.89 | 1 | 10 | 569 | 5.45 | 2.93 | 1 | 10 | 100 | 5.68 | 2.89 | 1 | 10 | 580 |
| No_Faith_in_Social_Mobility | 6.06 | 2.99 | 1 | 10 | 324 | 5.83 | 2.86 | 1 | 10 | 572 | 6.30 | 2.84 | 1 | 10 | 100 | 6.26 | 2.83 | 1 | 10 | 579 |
| Trust_in_State | 5.04 | 2.70 | 1 | 10 | 323 | 5.10 | 2.58 | 1 | 10 | 570 | 3.66 | 2.18 | 1 | 10 | 100 | 4.03 | 2.41 | 1 | 10 | 579 |
| Job_is_Secure | 0.70 | 0.46 | 0 | 1 | 324 | 0.68 | 0.47 | 0 | 1 | 572 | 0.73 | 0.45 | 0 | 1 | 100 | 0.74 | 0.44 | 0 | 1 | 580 |
| Right_Orientation | 0.39 | 0.49 | 0 | 1 | 324 | 0.33 | 0.47 | 0 | 1 | 572 | 0.27 | 0.45 | 0 | 1 | 100 | 0.24 | 0.43 | 0 | 1 | 580 |
| Religious | 0.52 | 0.50 | 0 | 1 | 324 | 0.52 | 0.50 | 0 | 1 | 572 | 0.46 | 0.50 | 0 | 1 | 100 | 0.53 | 0.50 | 0 | 1 | 580 |

**Note.** Table D3 provides a summary statistic of the control variables of our OLS analysis. A distinction is made between MB FLAT 1st GEN participants, MB NO FLAT 1st GEN participants, MB FLAT 2nd GEN participants, and MB NO FLAT 2nd GEN participants. This table provides information regarding the mean value (Mean), the standard deviation (SD), the minimum expression (Min.), and the maximum expression (Max.) of a variable. Moreover, the number of observations (N) is reported. All variables are defined as described in Appendix C, Table C1.

**Appendix E OLS Results**

**Table E1.** Hypotheses – OLS results with controls (corresponds to Table 2).

|  | Hypothesis 1 | Hypothesis 2a | Hypothesis 2b | Hypothesis 3a | Hypothesis 3b |
| --- | --- | --- | --- | --- | --- |
|  | Model 1 | Model 2 | Model 3 | Model 4 | Model 5 |
| Subsamples | NO_MB; MB | NO_MB; MB_NO_FLAT; MB_FLAT | | NO_MB; MB_1st_GEN; MB_2nd_GEN | |
| Reference group | NO_MB | MB_NO_FLAT | NO MB | MB_2nd_GEN | NO_MB |
| Constant | 14.448*** | 11.972*** | 14.437*** | 12.502*** | 13.781*** |
|  | (1.619) | (1.581) | (1.621) | (1.584) | (1.618) |
| MB | -2.768*** |  |  |  |  |
|  | (0.505) |  |  |  |  |
| NO_MB |  | 2.466*** |  | 1.279** |  |
|  |  | (0.536) |  | (0.633) |  |
| MB_FLAT |  | -1.116* | -3.582*** |  |  |
|  |  | (0.638) | (0.683) |  |  |
| MB_NO_FLAT |  |  | -2.466*** |  |  |
|  |  |  | (0.536) |  |  |
| MB_1st_GEN |  |  |  | -2.463*** | -3.742*** |
|  |  |  |  | (0.603) | (0.549) |
| MB_2nd_GEN |  |  |  |  | -1.279** |
|  |  |  |  |  | (0.633) |
| Age | 0.133*** | 0.132*** | 0.132*** | 0.146*** | 0.146*** |
|  | (0.018) | (0.018) | (0.018) | (0.018) | (0.018) |
| Female | -0.955* | -0.895* | -0.895* | -0.943* | -0.943* |
|  | (0.491) | (0.496) | (0.496) | (0.489) | (0.489) |
| Academic | 0.182 | 0.184 | 0.184 | 0.310 | 0.310 |
|  | (0.513) | (0.513) | (0.513) | (0.514) | (0.514) |
| Occupation_Status | -0.262 | -0.214 | -0.214 | -0.135 | -0.135 |
|  | (0.639) | (0.641) | (0.641) | (0.637) | (0.637) |
| Middle_Income | 0.001 | 0.005 | 0.005 | -0.121 | -0.121 |
|  | (0.574) | (0.574) | (0.574) | (0.573) | (0.573) |
| High_Income | -0.721 | -0.730 | -0.730 | -0.881 | -0.881 |
|  | (0.675) | (0.675) | (0.675) | (0.674) | (0.674) |
| Financial_Strain | -0.300*** | -0.300*** | -0.300*** | -0.308*** | -0.308*** |
|  | (0.109) | (0.109) | (0.109) | (0.109) | (0.109) |
| Risk_Attitude | 0.006 | 0.006 | 0.006 | -0.006 | -0.006 |
|  | (0.111) | (0.111) | (0.111) | (0.110) | (0.110) |
| Tax_Knowledge | -0.232 | -0.240 | -0.240 | -0.280 | -0.280 |
|  | (0.255) | (0.255) | (0.255) | (0.254) | (0.254) |
| Pro_Income_Equalization | 0.112 | 0.108 | 0.108 | 0.118 | 0.118 |
|  | (0.090) | (0.089) | (0.089) | (0.089) | (0.089) |
| No_Faith_in_Social_Mobility | 0.216** | 0.224*** | 0.224*** | 0.212** | 0.212** |
|  | (0.086) | (0.085) | (0.085) | (0.085) | (0.085) |
| Trust_in_State | 0.043 | 0.048 | 0.048 | 0.112 | 0.112 |
|  | (0.102) | (0.102) | (0.102) | (0.104) | (0.104) |
| Job_is_Secure | -0.227 | -0.282 | -0.282 | -0.311 | -0.311 |
|  | (0.672) | (0.673) | (0.673) | (0.671) | (0.671) |
| Right_Orientation | -2.643*** | -2.597*** | -2.597*** | -2.449*** | -2.449*** |
|  | (0.477) | (0.481) | (0.481) | (0.478) | (0.478) |
| Religious | -1.083** | -1.076** | -1.076** | -1.079** | -1.079** |
|  | (0.469) | (0.470) | (0.470) | (0.468) | (0.468) |
| N | 2,411 | 2,406 | 2,406 | 2,411 | 2,411 |
| Adjusted R-Squared | 0.068 | 0.069 | 0.069 | 0.075 | 0.075 |
| *** p ≤ 0.01; ** p ≤ 0.05; * p ≤ 0.10 | | | | | |

**Note*.*** Table E1 shows the same OLS results as presented in Table 2, but with all controls displayed. All variables are defined as described in Appendix C, Table C1.

**Table E2.** Further analysis – OLS results with controls (corresponds to Table 4).

|  | Model 1 | Model 2 | Model 3 | Model 4 |
| --- | --- | --- | --- | --- |
| Reference group | MB_FLAT_1st_GEN | MB_NO_FLAT_1st_GEN | MB_FLAT_2nd_GEN | MB_NO_FLAT_2ndGEN |
| Constant | 9.201*** | 10.501*** | 13.598*** | 12.237*** |
|  | (1.690) | (1.635) | (1.999) | (1.583) |
| NO_MB | 4.565*** | 3.265*** | 0.168 | 1.529** |
|  | (0.737) | (0.611) | (1.282) | (0.667) |
| MB_FLAT_1st_GEN |  | -1.300* | -4.397*** | -3.036*** |
|  |  | (0.759) | (1.380) | (0.782) |
| MB_NO_FLAT_1st_GEN | 1.300* |  | -3.097** | -1.736** |
|  | (0.759) |  | (1.317) | (0.695) |
| MB_FLAT_2nd_GEN | 4.397*** | 3.097** |  | 1.361 |
|  | (1.380) | (1.317) |  | (1.317) |
| MB_NO_FLAT_2nd_GEN | 3.036*** | 1.736** | -1.361 |  |
|  | (0.782) | (0.695) | (1.317) |  |
| Age | 0.143*** | 0.143*** | 0.143*** | 0.143*** |
|  | (0.018) | (0.018) | (0.018) | (0.018) |
| Female | -0.879* | -0.879* | -0.879* | -0.879* |
|  | (0.497) | (0.497) | (0.497) | (0.497) |
| Academic | 0.276 | 0.276 | 0.276 | 0.276 |
|  | (0.514) | (0.514) | (0.514) | (0.514) |
| Occupation_Status | -0.059 | -0.059 | -0.059 | -0.059 |
|  | (0.639) | (0.639) | (0.639) | (0.639) |
| Middle_Income | -0.076 | -0.076 | -0.076 | -0.076 |
|  | (0.576) | (0.576) | (0.576) | (0.576) |
| High_Income | -0.872 | -0.872 | -0.872 | -0.872 |
|  | (0.674) | (0.674) | (0.674) | (0.674) |
| Financial_Strain | -0.305*** | -0.305*** | -0.305*** | -0.305*** |
|  | (0.109) | (0.109) | (0.109) | (0.109) |
| Risk_Attitude | -0.007 | -0.007 | -0.007 | -0.007 |
|  | (0.111) | (0.111) | (0.111) | (0.111) |
| Tax_Knowledge | -0.268 | -0.268 | -0.268 | -0.268 |
|  | (0.254) | (0.254) | (0.254) | (0.254) |
| Pro_Income_Equalization | 0.115 | 0.115 | 0.115 | 0.115 |
|  | (0.089) | (0.089) | (0.089) | (0.089) |
| No_Faith_in_Social_Mobility | 0.220*** | 0.220*** | 0.220*** | 0.220*** |
|  | (0.085) | (0.085) | (0.085) | (0.085) |
| Trust_in_State | 0.114 | 0.114 | 0.114 | 0.114 |
|  | (0.104) | (0.104) | (0.104) | (0.104) |
| Job_is_Secure | -0.378 | -0.378 | -0.378 | -0.378 |
|  | (0.675) | (0.675) | (0.675) | (0.675) |
| Right_Orientation | -2.431*** | -2.431*** | -2.431*** | -2.431*** |
|  | (0.481) | (0.481) | (0.481) | (0.481) |
| Religious | -1.052** | -1.052** | -1.052** | -1.052** |
|  | (0.470) | (0.470) | (0.470) | (0.470) |
| N | 2,406 | 2,406 | 2,406 | 2,406 |
| Adjusted R-Squared | 0.075 | 0.075 | 0.075 | 0.075 |
| *** p ≤ 0.01; ** p ≤ 0.05; * p ≤ 0.10 | | | | |

**Note.** Table E2 shows the same OLS results as presented in Table 4, but with all controls displayed. All variables are defined as described in Appendix C, Table C1.

**Table E3.** Robustness check: Hypotheses – inclusion of Tax_Rate_Weak_Group.

|  | Hypothesis 1 | Hypothesis 2a | Hypothesis 2b | Hypothesis 3a | Hypothesis 3b |
| --- | --- | --- | --- | --- | --- |
|  | Model 1 | Model 2 | Model 3 | Model 4 | Model 5 |
| Subsamples | NO_MB; MB | NO_MB; MB_NO_FLAT; MB_FLAT | | NO_MB; MB_1st_GEN; MB_2nd_GEN | |
| Reference group | NO_MB | MB_NO_FLAT | NO MB | MB_2nd_GEN | NO_MB |
| Constant | 19.319*** | 17.050*** | 19.304*** | 17.537*** | 18.733*** |
|  | (1.561) | (1.519) | (1.564) | (1.512) | (1.568) |
| MB | -2.448*** |  |  |  |  |
|  | (0.455) |  |  |  |  |
| NO_MB |  | 2.254*** |  | 1.196** |  |
|  |  | (0.482) |  | (0.566) |  |
| MB_FLAT |  | -0.712 | -2.966*** |  |  |
|  |  | (0.555) | (0.603) |  |  |
| MB_NO_FLAT |  |  | -2.254*** |  |  |
|  |  |  | (0.482) |  |  |
| MB_1st_GEN |  |  |  | -2.074*** | -3.270*** |
|  |  |  |  | (0.538) | (0.496) |
| MB_2nd_GEN |  |  |  |  | -1.196** |
|  |  |  |  |  | (0.566) |
| Tax_Rate_Weak_Group | -0.633*** | -0.631*** | -0.631*** | -0.630*** | -0.630*** |
|  | (0.063) | (0.063) | (0.063) | (0.062) | (0.062) |
| Age | 0.114*** | 0.113*** | 0.113*** | 0.125*** | 0.125*** |
|  | (0.017) | (0.017) | (0.017) | (0.017) | (0.017) |
| Female | -1.964*** | -1.921*** | -1.921*** | -1.949*** | -1.949*** |
|  | (0.453) | (0.457) | (0.457) | (0.450) | (0.450) |
| Academic | 0.406 | 0.402 | 0.402 | 0.513 | 0.513 |
|  | (0.459) | (0.460) | (0.460) | (0.460) | (0.460) |
| Occupation_Status | -0.098 | -0.060 | -0.060 | 0.009 | 0.009 |
|  | (0.573) | (0.576) | (0.576) | (0.572) | (0.572) |
| Middle_Income | 0.069 | 0.090 | 0.090 | -0.034 | -0.034 |
|  | (0.521) | (0.521) | (0.521) | (0.520) | (0.520) |
| High_Income | -0.718 | -0.714 | -0.714 | -0.853 | -0.853 |
|  | (0.608) | (0.608) | (0.608) | (0.607) | (0.607) |
| Financial_Strain | -0.126 | -0.128 | -0.128 | -0.134 | -0.134 |
|  | (0.099) | (0.099) | (0.099) | (0.098) | (0.098) |
| Risk_Attitude | -0.005 | -0.005 | -0.005 | -0.016 | -0.016 |
|  | (0.099) | (0.099) | (0.099) | (0.099) | (0.099) |
| Tax_Knowledge | -0.025 | -0.035 | -0.035 | -0.067 | -0.067 |
|  | (0.230) | (0.230) | (0.230) | (0.230) | (0.230) |
| Pro_Income_Equalization | 0.092 | 0.089 | 0.089 | 0.098 | 0.098 |
|  | (0.081) | (0.081) | (0.081) | (0.080) | (0.080) |
| No_Faith_in_Social_Mobility | 0.220*** | 0.224*** | 0.224*** | 0.216*** | 0.216*** |
|  | (0.079) | (0.079) | (0.079) | (0.079) | (0.079) |
| Trust_in_State | 0.158* | 0.162* | 0.162* | 0.217** | 0.217** |
|  | (0.090) | (0.090) | (0.090) | (0.091) | (0.091) |
| Job_is_Secure | -0.494 | -0.531 | -0.531 | -0.563 | -0.563 |
|  | (0.606) | (0.610) | (0.610) | (0.608) | (0.608) |
| Right_Orientation | -2.191*** | -2.166*** | -2.166*** | -2.030*** | -2.030*** |
|  | (0.445) | (0.447) | (0.447) | (0.447) | (0.447) |
| Religious | -0.589 | -0.582 | -0.582 | -0.589 | -0.589 |
|  | (0.420) | (0.421) | (0.421) | (0.419) | (0.419) |
| N | 2,411 | 2,406 | 2,406 | 2,411 | 2,411 |
| Adjusted R-Squared | 0.252 | 0.251 | 0.251 | 0.256 | 0.256 |
| *** p ≤ 0.01; ** p ≤ 0.05; * p ≤ 0.10 | | | | | |

**Note.** Table E3 shows the OLS results from the robustness check. The structure of the regression models is analogous to the models presented in Table E1, with the difference that the variable Tax_Rate_Weak_Group is included as a control variable.

**Table E4.** Robustness check: Further analysis – inclusion of Tax_Rate_Weak_Group.

|  | Model 1 | Model 2 | Model 3 | Model 4 |
| --- | --- | --- | --- | --- |
| Reference group | MB FLAT 1st GEN | MB NO FLAT 1st GEN | MB FLAT 2nd GEN | MB NO FLAT 2nd GEN |
| Constant | 14.850*** | 15.755*** | 18.767*** | 17.247*** |
|  | (1.644) | (1.585) | (1.838) | (1.514) |
| NO_MB | 3.846*** | 2.942*** | -0.070 | 1.449** |
|  | (0.642) | (0.556) | (1.130) | (0.593) |
| MB_FLAT_1st_GEN |  | -0.905 | -3.917*** | -2.397*** |
|  |  | (0.660) | (1.195) | (0.682) |
| MB_NO_FLAT_1st_GEN | 0.905 |  | -3.012*** | -1.492** |
|  | (0.660) |  | (1.154) | (0.626) |
| MB_FLAT_2nd_GEN | 3.917*** | 3.012*** |  | 1.520 |
|  | (1.195) | (1.154) |  | (1.151) |
| MB_NO_FLAT_2nd_GEN | 2.397*** | 1.492** | -1.520 |  |
|  | (0.682) | (0.626) | (1.151) |  |
| Tax_Rate_Weak_Group | -0.629*** | -0.629*** | -0.629*** | -0.629*** |
|  | (0.063) | (0.063) | (0.063) | (0.063) |
| Age | 0.123*** | 0.123*** | 0.123*** | 0.123*** |
|  | (0.017) | (0.017) | (0.017) | (0.017) |
| Female | -1.901*** | -1.901*** | -1.901*** | -1.901*** |
|  | (0.457) | (0.457) | (0.457) | (0.457) |
| Academic | 0.481 | 0.481 | 0.481 | 0.481 |
|  | (0.461) | (0.461) | (0.461) | (0.461) |
| Occupation_Status | 0.076 | 0.076 | 0.076 | 0.076 |
|  | (0.576) | (0.576) | (0.576) | (0.576) |
| Middle_Income | 0.020 | 0.020 | 0.020 | 0.020 |
|  | (0.521) | (0.521) | (0.521) | (0.521) |
| High_Income | -0.838 | -0.838 | -0.838 | -0.838 |
|  | (0.607) | (0.607) | (0.607) | (0.607) |
| Financial_Strain | -0.134 | -0.134 | -0.134 | -0.134 |
|  | (0.098) | (0.098) | (0.098) | (0.098) |
| Risk_Attitude | -0.016 | -0.016 | -0.016 | -0.016 |
|  | (0.099) | (0.099) | (0.099) | (0.099) |
| Tax_Knowledge | -0.060 | -0.060 | -0.060 | -0.060 |
|  | (0.230) | (0.230) | (0.230) | (0.230) |
| Pro_Income_Equalization | 0.095 | 0.095 | 0.095 | 0.095 |
|  | (0.080) | (0.080) | (0.080) | (0.080) |
| No_Faith_in_Social_Mobility | 0.221*** | 0.221*** | 0.221*** | 0.221*** |
|  | (0.079) | (0.079) | (0.079) | (0.079) |
| Trust_in_State | 0.219** | 0.219** | 0.219** | 0.219** |
|  | (0.091) | (0.091) | (0.091) | (0.091) |
| Job_is_Secure | -0.615 | -0.615 | -0.615 | -0.615 |
|  | (0.612) | (0.612) | (0.612) | (0.612) |
| Right_Orientation | -2.023*** | -2.023*** | -2.023*** | -2.023*** |
|  | (0.448) | (0.448) | (0.448) | (0.448) |
| Religious | -0.562 | -0.562 | -0.562 | -0.562 |
|  | (0.420) | (0.420) | (0.420) | (0.420) |
| N | 2,406 | 2,406 | 2,406 | 2,406 |
| Adjusted R-Squared | 0.256 | 0.256 | 0.256 | 0.256 |
| *** p ≤ 0.01; ** p ≤ 0.05; * p ≤ 0.10 | | | | |

**Note.** Table E4 shows the OLS results from the robustness check. The structure of the regression models is analogous to the models presented in Table E2, with the difference that the variable Tax_Rate_Weak_Group is included as a control variable.

**Table E5.** Robustness check: Hypotheses – exclusion of control variables.

|  | Hypothesis 1 | Hypothesis 2a | Hypothesis 2b | Hypothesis 3a | Hypothesis 3b |
| --- | --- | --- | --- | --- | --- |
|  | Model 1 | Model 2 | Model 3 | Model 4 | Model 5 |
| Subsamples | NO_MB; MB | NO_MB; MB_NO_FLAT; MB_FLAT | | NO_MB; MB_1st_GEN; MB_2nd_GEN | |
| Reference group | NO_MB | MB_NO_FLAT | NO MB | MB_2nd_GEN | NO_MB |
| Constant | 17.392*** | 14.152*** | 17.392*** | 14.749*** | 17.392*** |
|  | (0.402) | (0.333) | (0.402) | (0.450) | (0.402) |
| MB | -3.653*** |  |  |  |  |
|  | (0.492) |  |  |  |  |
| NO_MB |  | 3.240*** |  | 2.642*** |  |
|  |  | (0.522) |  | (0.603) |  |
| MB_FLAT |  | -1.487** | -4.727*** |  |  |
|  |  | (0.636) | (0.675) |  |  |
| MB_NO_FLAT |  |  | -3.240*** |  |  |
|  |  |  | (0.522) |  |  |
| MB_1st_GEN |  |  |  | -1.777*** | -4.419*** |
|  |  |  |  | (0.577) | (0.541) |
| MB_2nd_GEN |  |  |  |  | -2.642*** |
|  |  |  |  |  | (0.603) |
| N | 2,422 | 2,416 | 2,416 | 2,422 | 2,422 |
| Adjusted R-Squared | 0.022 | 0.024 | 0.024 | 0.026 | 0.026 |
| *** p ≤ 0.01; ** p ≤ 0.05; * p ≤ 0.10 | | | | | |

**Note.** Table E5 shows the OLS results from the robustness check. The structure of the regression models is analogous to the models presented in Table E1, with the difference that control variables are excluded.

**Table E6.** Robustness check: Further analysis – exclusion of control variables.

|  | Model 1 | Model 2 | Model 3 | Model 4 |
| --- | --- | --- | --- | --- |
| Reference group | MB FLAT 1st GEN | MB NO FLAT 1st GEN | MB FLAT 2nd GEN | MB NO FLAT 2nd GEN |
| Constant | 11.648*** | 13.759*** | 15.960*** | 14.540*** |
|  | (0.591) | (0.457) | (1.228) | (0.485) |
| NO_MB | 5.744*** | 3.633*** | 1.432 | 2.852*** |
|  | (0.714) | (0.608) | (1.292) | (0.630) |
| MB_FLAT_1st_GEN |  | -2.111*** | -4.312*** | -2.892*** |
|  |  | (0.747) | (1.363) | (0.764) |
| MB_NO_FLAT_1st_GEN | 2.111*** |  | -2.201* | -0.781 |
|  | (0.747) |  | (1.310) | (0.666) |
| MB_FLAT_2nd_GEN | 4.312*** | 2.201* |  | 1.420 |
|  | (1.363) | (1.310) |  | (1.320) |
| MB_NO_FLAT_2nd_GEN | 2.892*** | 0.781 | -1.420 |  |
|  | (0.764) | (0.666) | (1.320) |  |
| N | 2,416 | 2,416 | 2,416 | 2,416 |
| Adjusted R-Squared | 0.028 | 0.028 | 0.028 | 0.028 |
| *** p ≤ 0.01; ** p ≤ 0.05; * p ≤ 0.10 | | | | |

**Note.** Table E6 shows the OLS results from the robustness check. The structure of the regression models is analogous to the models presented in Table E2, with the difference that control variables are excluded.

**Appendix F** **Overview of the distribution of tax rate levels**

**Table F1.** Tax level distribution by subsample.

|  | **Distribution of the tax rate level for the weak group (in %)** | | | | | | | | | | | |
| --- | --- | --- | --- | --- | --- | --- | --- | --- | --- | --- | --- | --- |
| **Tax rate level** | **0%** | | **5%** | | **10%** | | **15%** | | **20%** | | **Other** | |
| **NO_MB** | 2.9 | | 28.1 | | 36.4 | | 6.2 | | 2.9 | | 23.5 | |
| **MB_NO_FLAT** | 2.7 | | 29.2 | | 31.8 | | 6.3 | | 3.2 | | 26.8 | |
| **MB_FLAT** | 0.9 | | 24.3 | | 34.9 | | 10.4 | | 1.4 | | 28.1 | |
| **MB_1st_GEN** | 3.0 | | 28.8 | | 34.6 | | 6.2 | | 3.2 | | 24.2 | |
| **MB_2nd_GEN** | 3.1 | | 29.6 | | 32.4 | | 6.3 | | 3.5 | | 25.1 | |
|  | **Distribution of the tax rate level for the strong group (in %)** | | | | | | | | | | | |
| **Tax rate level** | **10%** | **15%** | | **20%** | | **25%** | | **30%** | | **35%** | | **Other** |
| **NO_MB** | 5.5 | 7.3 | | 14.8 | | 19.8 | | 23.3 | | 8.7 | | 20,6 |
| **MB_NO_FLAT** | 9.2 | 10.5 | | 17.8 | | 18.7 | | 16.2 | | 7.1 | | 20.5 |
| **MB_FLAT** | 9.2 | 13.4 | | 15.3 | | 21.2 | | 16.3 | | 6.4 | | 18.2 |
| **MB_1st_GEN** | 6.8 | 9.5 | | 15.4 | | 18.3 | | 20.9 | | 8.5 | | 20.6 |
| **MB_2nd_GEN** | 8.5 | 12.2 | | 16.3 | | 16.4 | | 17.9 | | 8.2 | | 20.5 |

1. The initial level of the personal tax rate depended on a participant’s assigned individual financial status; i.e., the tax rate was either 25% or 10%. [↑](#footnote-ref-1)
